# Metabolites With Cytotoxic Activities From the Mangrove Endophytic Fungus *Fusarium* sp. 2ST2

**DOI:** 10.3389/fchem.2022.842405

**Published:** 2022-02-15

**Authors:** Yan Chen, Guisheng Wang, Yilin Yuan, Ge Zou, Wencong Yang, Qi Tan, Wenyi Kang, Zhigang She

**Affiliations:** ^1^ National R & D Center for Edible Fungus Processing Technology, Henan University, Kaifeng, China; ^2^ School of Chemistry, Sun Yat-sen University, Guangzhou, China

**Keywords:** mangrove, endophytic fungus, *Fusarium* sp., cytotoxicity, benzofuran, chromone

## Abstract

Two new 3-decalinoyltetramic acid derivatives with peroxide bridge fusarisetins E (**1**) and F (**2**), one new chromone fusarimone A (**5**), two new benzofurans fusarifurans A (**9**) and B (**10**), three new isocoumarins fusarimarins A–C (**11–13**), as well as five known analogues **3**, **4**, **6–8** and **14** were isolated from mangrove endophytic fungus *Fusarium* sp. 2ST2. Their structures and absolute configurations were established by spectroscopic analysis, density functional theory-gauge invariant atomic orbital NMR calculation with DP4+ statistical analysis, and electronic circular dichroism calculation. Compounds **1** and **2** showed significant cytotoxicity against human A549 cell lines with IC_50_ values of 8.7 and 4.3 μM, respectively.

## Introduction

Endophytic fungi, inhabiting plants without any negative effects for the host, have been proven to be a promising source of novel structures and unique bioactivities (Liu et al., 2021; Viridiana et al., 2021). *Fusarium* spp. are endophytic fungi widely distributed in association with plants. It has attracted much attention due to their diverse bioactive secondary metabolites, including alkaloids, terpenes, cyclopeptide, anthraquinone, and lactones (Chen et al., 2019)—for example, indole alkaloids fusaindoterpenes A and B from *Fusarium* sp. showed antiviral activity (Guo et al., 2020), and fusarithioamide A from *Fusarium chlamydosporium* exhibited cytotoxic activity (Ibrahim et al., 2018).

**FIGURE 1:**
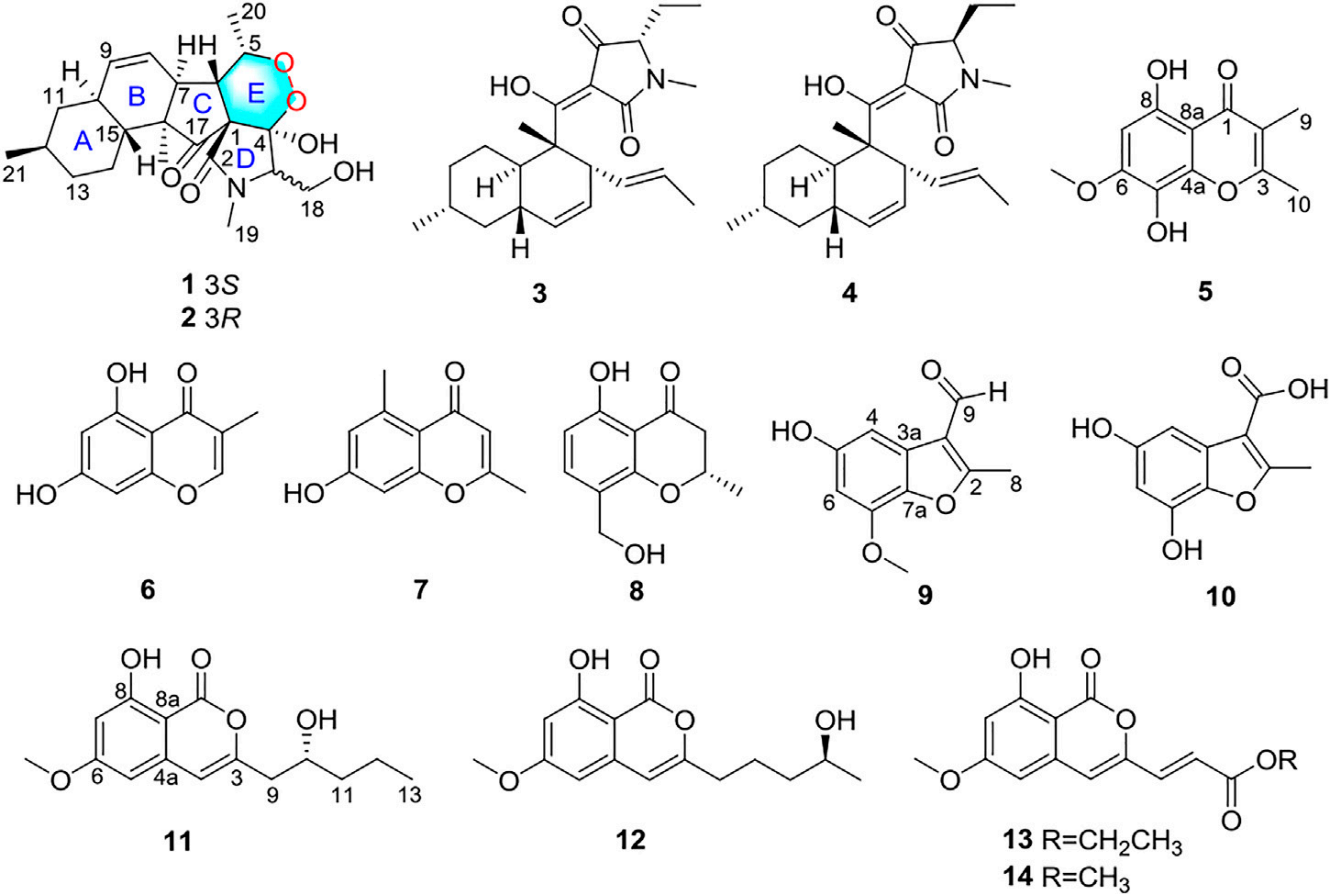
Structures of compounds **1**–**14**.

Mangrove endophytic fungi, the second largest ecological group of marine fungi, have been reported to produce thousands of new metabolites until now (Chen et al., 2021; Chen et al., 2022). Over the past 2 decades, our group continues to explore bioactive novel structures from mangrove endophytic fungi (Huang et al., 2013; Xiao et al., 2013; Liu et al., 2016; Cui et al., 2017; Cai et al., 2019). In the course of our ongoing search for new antitumor active compounds from mangrove endophytic fungi, the strain *Fusarium* sp. 2ST2 attracted our attention because of the cytotoxicity of the crude extract. Then, eight new metabolites, including two alkaloids fusarisetin E (**1**) and F (**2**), one chromone fusarimone A (**5**), two benzofurans fusarifurans A (**9**) and B (**10**), three isocoumarins fusarimarins A–C (**11**–**13**), were obtained together with five analogues equisetin (**3**), epi-equisetin (**4**), takanechromone B (**6**), altechromone A (**7**), 4H-1-benzopyran-4-one-2,3-dihydro-5-hydroxy-8-(hydroxylmethyl)-2-methyl (**8**), and aspergisocoumrin A (**14**) (Figure 1). As expected, compounds **1** and **2** exhibited significant cytotoxicity against human A549 cell line, and compounds **8** and **14** showed potent cytotoxicity against A549 and MDA-MB-435 cell lines. The isolation, structure elucidation, and biological evaluation of these compounds were reported herein.

## Materials and Methods

### General Experimental Procedures

Optical rotations were measured on a PerkinElmer 341 instrument at 25°C. Melting points were recorded on a Fisher-Johns hot-stage apparatus. UV spectra were measured in MeOH using a Shimadzu UV-2700 spectrophotometer. Electronic circular dichroism (ECD) data were obtained on a Chirascan CD spectrometer (Applied Photophysics). A Bruker Avance 500 spectrometer (^1^H 500 MHz, ^13^C 125 MHz) was used for the 1D and 2D NMR data collection. All high-resolution electrospray ionization mass spectrometry (HRESIMS) data were obtained on an Agilent G6230 Q-TOF mass spectrometer. Silica gel (200–300 mesh, Qingdao Marine Chemical Factory) and Sephadex LH-20 (Amersham Pharmacia) were used in the column chromatography (CC). Silica gel plates (Qingdao Huang Hai Chemical Group Co., G60, F-254) were used for the thin-layer chromatography.

### Fungal Material

The fungus *Fusarium* sp. 2ST2 was isolated from healthy leaves of *Kandelia candel*, which was collected in June 2015 from the South China Sea, Dong Zhai Harbor Mangrove Nature Reserve Area, Hainan Province, China. The strain was identified as *Fusarium* sp. (GenBank no. MZ801734) by a BLAST search which showed it to be 100% identical with the sequence of *Fusarium* sp. (GenBank no. KU296944.1).

### Fermentation, Extraction, and Isolation

The fungus *Fusarium* sp. 2ST2 was cultivated on potato dextrose agar for 5 days. The mycelia of the strain were inoculated into 500 ml potato dextrose broth for 3 days to prepare the seed culture and then inoculated into the solid rice medium (70 g of rice, 3 g peptone, and 50 ml of distilled water, 60 flasks). It was incubated for 30 days at room temperature.

The medium was extracted with MeOH for three times, and the total residue of the strain (65.0 g) was obtained. The EtOAc extract was chromatographed by silica gel CC (200–300 mesh silica) and eluted with an increasing gradient of petroleum ether/EtOAc (9:1 to 1:9) to afford six fractions (Fr. A–F). Fraction B was applied to Sephadex LH-20 CC (CH_2_Cl_2_/MeOH v/v, 1:1) to give three fractions (Fr. B1–B3). Fraction B1 was subjected to silica gel CC (CH_2_Cl_2_/MeOH v/v, 98:2) to yield compounds **3** (5.8 mg) and **14** (2.5 mg). Fraction B2 was subjected to silica gel CC (CH_2_Cl_2_/MeOH v/v, 96:4) to yield compounds **9** (8.6 mg) and **13** (4.3 mg). Fraction C was eluted on Sephadex LH-20 CC (100% MeOH) to obtain compound **10** (7.5 mg) and two other fractions (Fr. C1–C2). Fraction C2 was separated using silica gel CC (CH_2_Cl_2_/MeOH v/v, 95:5) to yield compounds **11** (3.1 mg) and **12** (3.5 mg). Fraction D was eluted on Sephadex LH-20 CC (100% MeOH) to afford three fractions (Fr. D1–D3). Fraction D1 was purified by semipreparative UPLC (MeOH-H_2_O, 7:3) to give compounds **1** (3.6 mg) and **2** (4.0 mg). Fraction D2 was subjected to silica gel CC (CH_2_Cl_2_/MeOH v/v, 9:1) to give compounds **4** (2.5 mg) and **7** (6.5 mg). Fraction E was subjected to Sephadex LH-20 CC (CH_2_Cl_2_/MeOH v/v, 1:1) to give compound **6** (3.8 mg) and another fraction E1. Compounds **5** (3.0 mg) and **8** (2.8 mg) were obtained from fraction E1, which was subjected to UPLC (MeOH-H_2_O, 6:4).

“Fusarisetin E (**1**): Colorless oil, [α] + 10.0 (*c* = 0.16, MeOH). UV (MeOH) *λ*_max_ (log *ε*): 206 (3.02), 280 (2.16) nm. HRESIMS *m/z* 406.22293 [M + H]^+^ (calculated for C_22_H_32_NO_6_ 406.22241). ^1^H and ^13^C NMR (CD_3_OD-*d*_4_) data, see Table 1.

**TABLE 1:** ^1^H and ^13^C NMR data of compounds **1** and **2**.

No.	1			2		
	*δ*_C_ ^a^	*δ*_H_ ^a^	*δ*_H_ ^b^	*δ*_C_ ^a^	*δ*_H_ ^a^	*δ*_H_ b
1	65.1			65.3		
2	171.2			172.1		
3	68.9	3.16, dd (5.3, 6.8)	3.0, dd (4.2, 7.8)	68.6	4.0, dd (2.7, 7.4)	3.82, brd (8.5)
4	103.2			103.2		
5	76.8	4.37, qd (3.1, 6.9)	4.26, m	76.7	4.36, qd (3.2, 6.9)	4.23, m
6	46.2	2.59, dd (3.1, 11.9)	2.73, dd (3.6, 11.7)	45.1	2.59, dd (3.1, 11.9)	2.76, brd (9.5)
7	44.6	2.85, dd (4.7, 11.9)	2.44, dd (2.2, 11.9)	44.4	2.86, dd (4.6, 11.8)	2.44, dd (2.2, 12.0)
8	127.7	5.89, ddd (2.4, 4.8, 10.1)	5.84, brd (8.8)	127.7	5.88, ddd (2.4, 4.8, 10.1)	5.83, brd (8.8)
9	133.8	5.56, brd (10.1)	5.52, brd (10.1)	133.7	5.56, brd (10.1)	5.52, brd (9.8)
10	38.4	1.89, m	1.84, m	38.3	1.88, m	1.84, m
11	43.0	1.87, m	1.81, m	43.1	1.86, m	1.81, m
		0.82, q (12.7)	0.73, q (12.7)		0.82, q (12.7)	0.73, q (12.7)
12	34.1	1.49, m	1.41, m	34.1	1.49, m	1.41, m
13	36.5	1.75, m	1.70, m	36.4	1.75, m	1.70, m
		0.91, m	0.85, m		0.90, m	0.85, m
14	26.3	1.56, m	1.45, m	26.3	1.55, m	1.45, m
		1.0, m	1.04, m		1.0, m	1.04, m
15	40.0	1.37, m	1.18, m	39.8	1.37, m	1.18, m
16	53.2			53.1		
17	214.3			213.5		
18	62.6	4.07, dd (6.9, 11.7)	3.88, m	58.5	3.95, dd (2.8, 12.3)	3.87, brd (12.1)
		3.96, dd (5.3, 11.7)	3.78, dd (3.1, 11.0)		3.66, dd (7.4, 12.3)	3.38, m
19	30.5	3.03, s	2.92, s	28.6	3.02, s	2.88, s
20	17.7	1.34, d (7.0)	1.26, d (6.9)	17.4	1.33, d (7.0)	1.24, d (6.1)
21	22.7	0.93, d (6.5)	0.86, d (6.5)	22.7	0.93, d (6.5)	0.87, d (6.3)
22	15.0	1.0, s	0.89, s	14.7	1.0, s	0.91, s
OH-4			4.88, s			4.9, s
OH-18			7.3, s			7.5, s

a Measured in CD_3_OD.

b Measured in DMSO-*d*_6_.

Fusarisetin E (**2**): Colorless oil, [α] + 11.2 (*c* = 0.19, MeOH). UV (MeOH) *λ*_max_ (log *ε*): 204 (3.0), 282 (2.56) nm. HRESIMS *m/z* 406.22274 [M + H]^+^ (calculated for C_22_H_32_NO_6_ 406.22241). ^1^H and ^13^C NMR (CD_3_OD*-d*_4_) data, see Table 1.

Fusarimone A (**5**): Yellow solid. HRESIMS *m/z* 237.07583 [M + H]^+^ (calculated for C_12_H_13_O_5_ 237.07575). ^1^H and ^13^C NMR (CDCl_3_) data, see Table 2.

**TABLE 2:** ^1^H and ^13^C NMR data of compound **5** in CDCl_3_.

5			5		
No.	*δ*_C_	*δ*_H_	No.	*δ*_C_	*δ*_H_
1	182.2		8	154.3	
2	114.7		8a	104.2	
3	162.9		9	9.2	2.02, s
4a	143.3		10	18.6	2.44, s
5	125.2		OCH_3_-6	56.5	3.96, s
6	151.4		OH-5		5.14, s
7	94.5	6.41, s	OH-8		12.51, s

Fusarifuran A (**9**): White solid, HRESIMS *m/z* 205.05090 [M-H]- (calculated for C_11_H_9_O_4_ 205.05063). ^1^H and ^13^C NMR (CD_3_OD*-d*_4_) data, see Table 3.

**TABLE 3:** ^1^H and ^13^C NMR data of **9** and **10** in CD_3_OD.

No.	9		10	
	*δ*_C_	*δ*_H_ (*J* in Hz)	*δ*_C_	*δ*_H_ (*J* in Hz)
2	169.4		163.5	
3	119.1		128.4	
3a	127.6		109.0	
4	98.9	7.02, d (2.2)	99.6	6.84, d (2.3)
5	139.1		137.2	
6	98.6	6.44, d (2.2)	97.3	6.27, d (2.3)
7	146.5		141.8	
7a	156.9		154.3	
8	12.7	2.75, s	13.1	2.70, s
9	187.4	10.14, s	166.3	
10	56.5	3.95, s		

Fusarifuran B (**10**): White solid, HRESIMS *m/z* 207.03026 [M-H]^‒^ (calculated for C_10_H_7_O_5_ 207.02990). ^1^H and ^13^C NMR (CD_3_OD*-d*_4_) data, see Table 3.

Fusarimarin A (**11**): Colorless oil, [α] −21.5 (*с* 0.06, MeOH). UV (MeOH) *λ*_max_ (log *ε*): 219 (3.2), 238 (2.4), 318 (3.5) nm. HRESIMS *m/z* 279.12288 [M + H]^+^ (calculated for C_15_H_19_O_5_ 279.12270). ^1^H and ^13^C NMR (CDCl_3_) data, see Table 4.

**TABLE 4:** ^1^H and^13^C NMR data of **11–13** in CDCl_3_.

No.	11		12		13	
	*δ*_C_	*δ*_H_ (*J* in Hz)	*δ*_C_	*δ*_H_ (*J* in Hz)	*δ*_C_	*δ*_H_ (*J* in Hz)
1	166.2		166.6		164.8	
3	155.0		157.7		149.8	
4	106.1	6.29, s	104.3	6.19, s	111.8	6.60, s
4a	139.1		139.5		134.3	
5	101.4	6.33, d (2.2)	100.4	6.46, d (2.2)	103.6	6.49, brs
6	166.9		166.9		166.8	
7	100.5	6.48, d (2.2)	101.3	6.31, d (2.2)	102.1	6.58, brs
8	163.6		163.8		163.9	
8a	100.0		100.1		102.1	
9	41.6	2.69, dd (3.7, 14.6)	33.3	2.53, t (2.5)	134.3	7.22, d (15.5)
		2.55, dd (8.6, 14.6)				
10	68.9	4.07, m	23.2	1.83, m	122.5	6.68, d (15.5)
				1.73, m		
11	39.3	1.53, m	38.5	1.52, m	166.1	
12	18.7	1.51, m	67.9	3.84, m	61.0	4.29, dd (7.1, 14.1)
13	14.0	0.96, t (6.9)	23.9	1.22, d (6.2)	14.2	1.36, t (7.0)
OH-8		11.10, s		11.10, s		11.0, s
OCH_3_-6	55.7	3.87, s	55.8	3.86, s	55.9	3.91, s

Fusarimarin B (**12**): Colorless oil, [α] +18.6 (*с* 0.07, MeOH). UV (MeOH) *λ*_max_ (log *ε*): 220 (3.3), 252 (3.0), 316 (3.4) nm. HRESIMS *m/z* 279.12290 [M + H]^+^ (calculated for C_15_H_19_O_5_ 279.12270). ^1^H and ^13^C NMR (CDCl_3_) data, see Table 4.

Fusarimarin C (**13**): Colorless oil, HRESIMS *m/z* 291.08639 [M + H]^+^ (calculated for C_15_H_15_O_6_ 291.08631). ^1^H and ^13^C NMR (CDCl_3_) data, see Table 4.

### NMR Calculations

In general, conformational analysis was carried out using Merck Molecular Field by Spartan’s 10 software. Conformers above 1% Boltzmann populations were optimized at the B3LYP/6-311+G (d, p) level in polarizable continuum model (PCM) methanol (Gaussian 09). Subsequently, NMR calculations were computed using the gauge invariant atomic orbital (GIAO) method at the mPWLPW91-SCRF/6-311+G (d, p) level using the PCM in methanol (Gaussian 09). Finally, the shielding constants were averaged by Boltzmann distribution theory for each stereoisomer, and their experimental and calculation data were analyzed by DP4+ probability.

### ECD Calculations

The ECD calculations were performed as described previously (Chen et al., 2020). Geometric optimization of compounds **1** and **2** was carried out at the B3LYP/6-31+G(d) level in the liquid phase. Then, ECD calculations were performed using the TDDFT methodology at the WB97XD/CC-PVDZ and WB97XD/6-31G levels, respectively.

### Cytotoxicity Assay

The cytotoxicity of all compounds against tumor cell lines was tested by the MTT assay as previously reported (Chen et al., 2019).

## Results and Discussion

**FIGURE 2:**
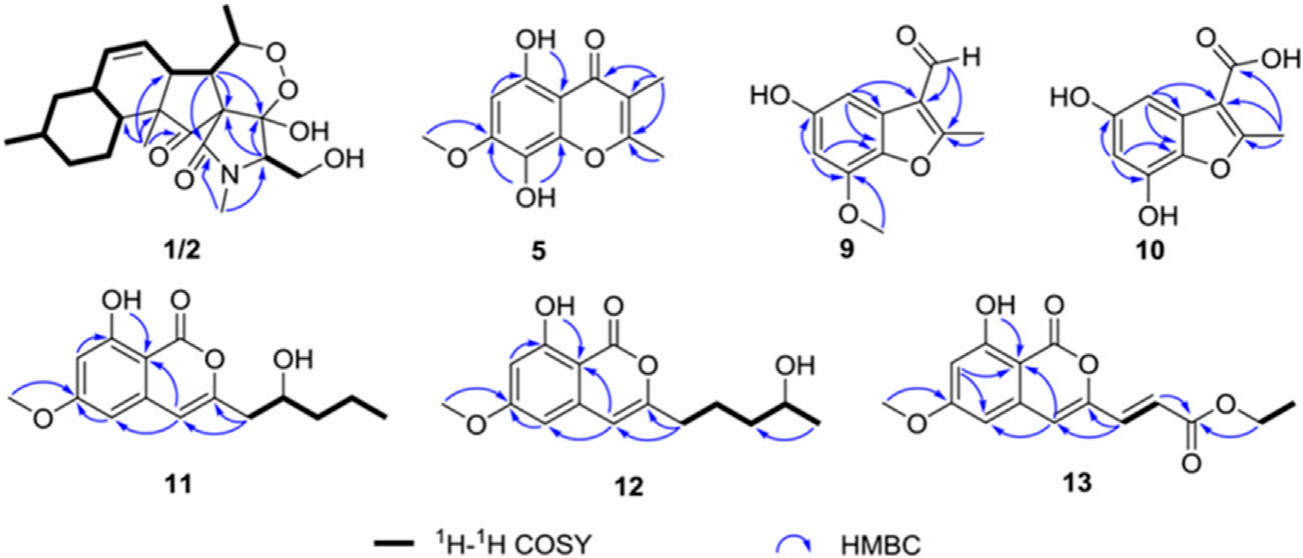
Key heteronuclear multiple-bond correlations and correlation spectroscopy of compounds **1**, **2**, **5** and **9–13**.

Compound **1** was isolated as a colorless oil. The molecular formula was determined as C_22_H_32_NO_6_ based on the HRESIMS data (*m/z* 406.22293 [M + H]^+^). The ^1^H NMR data of **1** (Table 1) showed three methyl signals at *δ*_H_ 0.93 (d, *J* = 6.5 Hz), 1.0 (s), and 1.34 (d, *J* = 7.0 Hz), one N-methyl proton at *δ*_H_ 3.03 (s), and two olefinic protons at *δ*_H_ 5.56 (brd, *J* = 10.1 Hz) and 5.89 (ddd, *J* = 2.4, 4.8, 10.1 Hz). The ^13^C NMR data of **1** (Table 1) displayed 22 carbon signals, including four methyls, four methylenes (one oxygenated), seven methines (two olefinic), and four quaternary carbons (one ketone carbonyl and one ester carbonyl carbon). The planar structure of **1** was a detailed analysis of the 1D and 2D NMR data. The spin system of H_3_-20/H-5/H-6/H-7/H-8/H-9/H-10/H_2_-11/H-12(/H_3_-21)/H_2_-13/H_2_-14/H-15(/H-10) from ^1^H-^1^H COSY data (Figure 2), together with the heteronuclear multiple-bond correlations (HMBC) (Figure 2) from H_3_-22 to C-7, C-15, C-16 and C-17 and from H-6 to C-1, established the partial ring system of A/B/C, while the HMBC correlations from H-6 to C-2 and C-4, from H-3 to C-1 and C-4, and from H_3_-19 to C-2 and C-3 indicated the presence of a *γ*-lactam moiety (ring D). In addition, a peroxide bridge between C-4 and C-5 was proposed according to two additional oxygen atoms in the molecular formula of **1**, which constitute the ring E. Thus, the planar structure of **1** was deduced (Figure 1), which was similar to fusarisetin A (Jang et al., 2011), by comparing their NMR data.

Compound **2**, with a molecular formula of C_22_H_32_NO_6_, the same as **1**, was isolated as a colorless oil. The results of comparing the NMR data of **1** and **2** indicated that they shared a planar structure, and this was further confirmed by an extensive analysis of ^1^H-^1^H COSY and HMBC correlations (Figure 2), while the major difference of NMR shifts at H-3 (*Δδ*_H_ +0.84), C-18 (*Δδ*_C_ −4.1), and C-19 (*Δδ*_C_ −1.9) suggested **1** and **2** to be 3-epimers.

**FIGURE 3:**
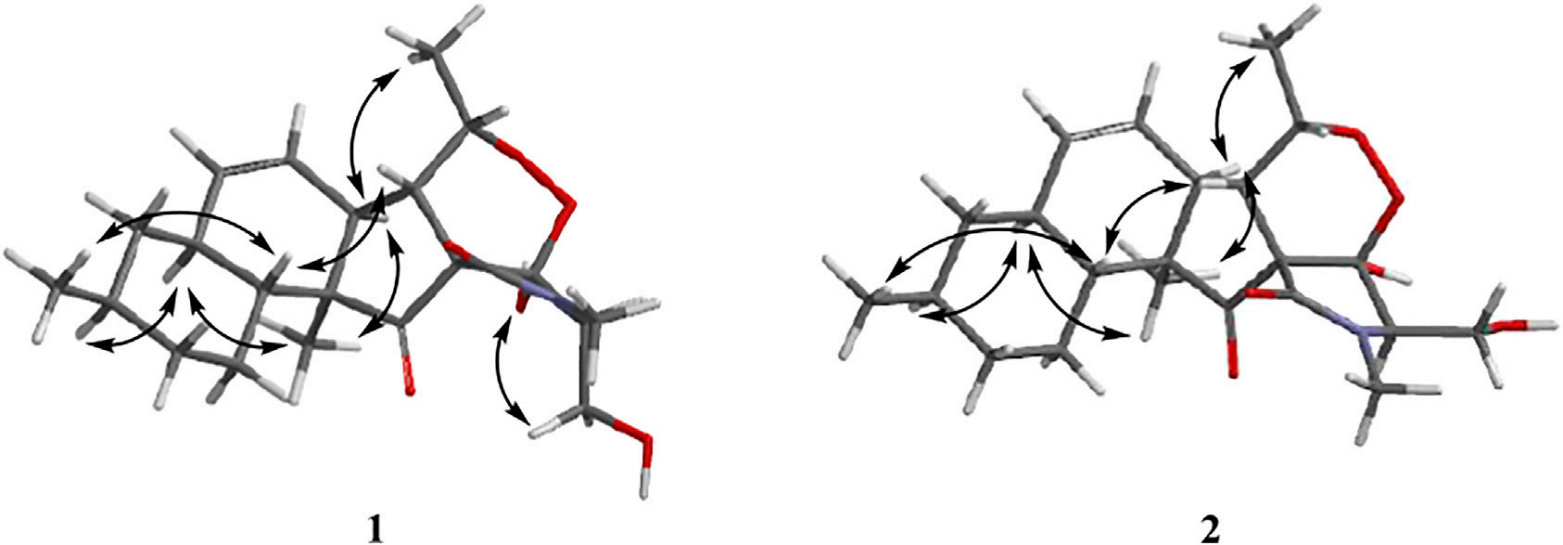
Key nuclear Overhauser effect correlations of compounds **1** and **2**.

**FIGURE 4:**
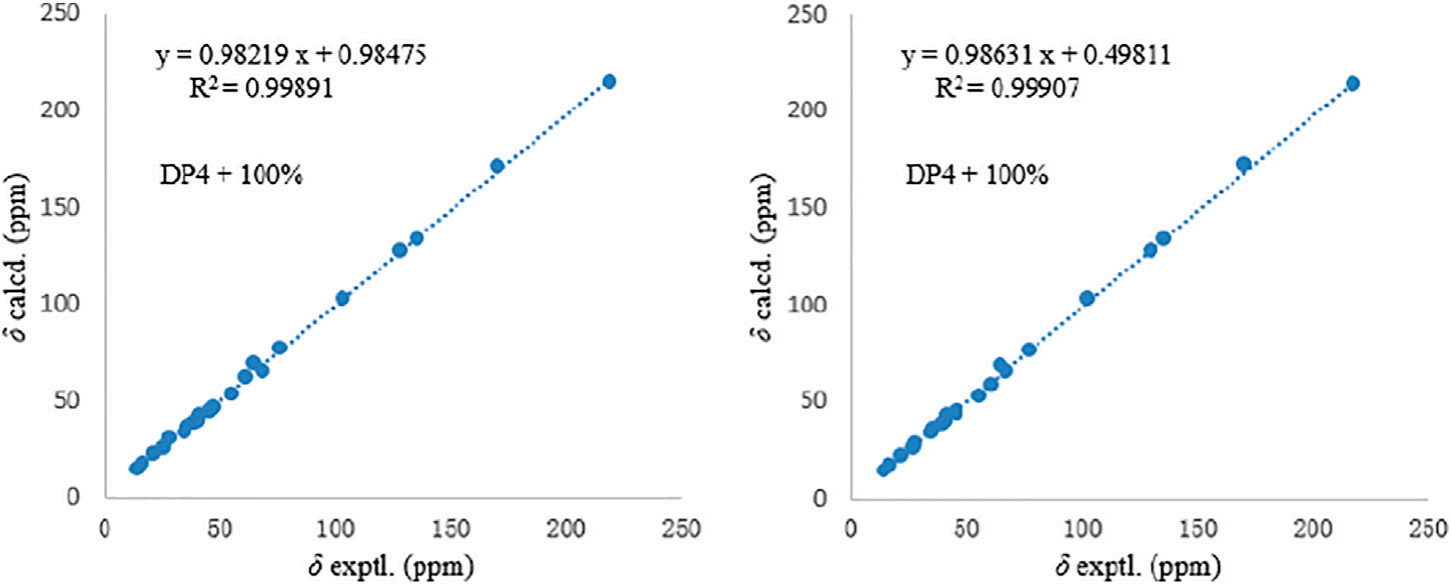
Comparisons of calculated and experimental ^13^C NMR data of **1** and **2**.

The relative configurations of **1** and **2** were determined by the NOESY correlations (Figure 3). The cross-peaks of H-12/H-10/H_3_-22/H-7/H_3_-20 suggested that these protons were co-facial, while the correlations of H_3_-21/H-15/H-6 showed that these protons were on the other face. Considering the absence of correlation from H_2_-18 and 4-OH to other protons, the NOESY spectrum of **1** and **2** were retested in DMSO-*d*_6_ reagent. Then, the correlation of 4-OH/H_2_-18 was only detected in **1**, indicating that the protons of OH-4 and H_2_-18 were positioned on the same face in **1** and were opposite in **2**. Thus, **1** and **2** were an epimer at C-3. Subsequently, the ^13^C NMR calculations of (1R*, 3S*, 4R*, 5S*, 6S*, 7S*, 10S*, 21R*, 15R*, 16S*)**-1a** and (1R*, 3R*, 4S*, 5S*, 6S*, 7S*, 10S*, 21R*, 15R*, 16S*)**-1b** were carried out using the GIAO method at mPW1PW91-SCRF/6–311+G (d, p)/PCM (MeOH). The results of the DP4+ probability analysis (Smith and Goodman, 2010; Kawazoe et al., 2020; Xu et al., 2021) showed that **1a** was the most likely candidate structure, with a better correlation coefficient (*R* ^2^ = 0.99891) and a high DP4+ probability of 100% (all data) probability (Figure 4). Similarly, ^13^C NMR calculations with the DP4+ probability analysis of the two isomers [(1R*, 3S*, 4S*, 5S*, 6S*, 7S*, 10S*, 21R*, 15R*, 16S*)**-2a** and (1R*, 3R*, 4R*, 5S*, 6S*, 7S*, 10S*, 21R*, 15R*, 16S*)-**2b**] of **2** were performed. The results showed that **2** gave the best match of 100% (all data) with the **2b** isomer.

**FIGURE 5:**
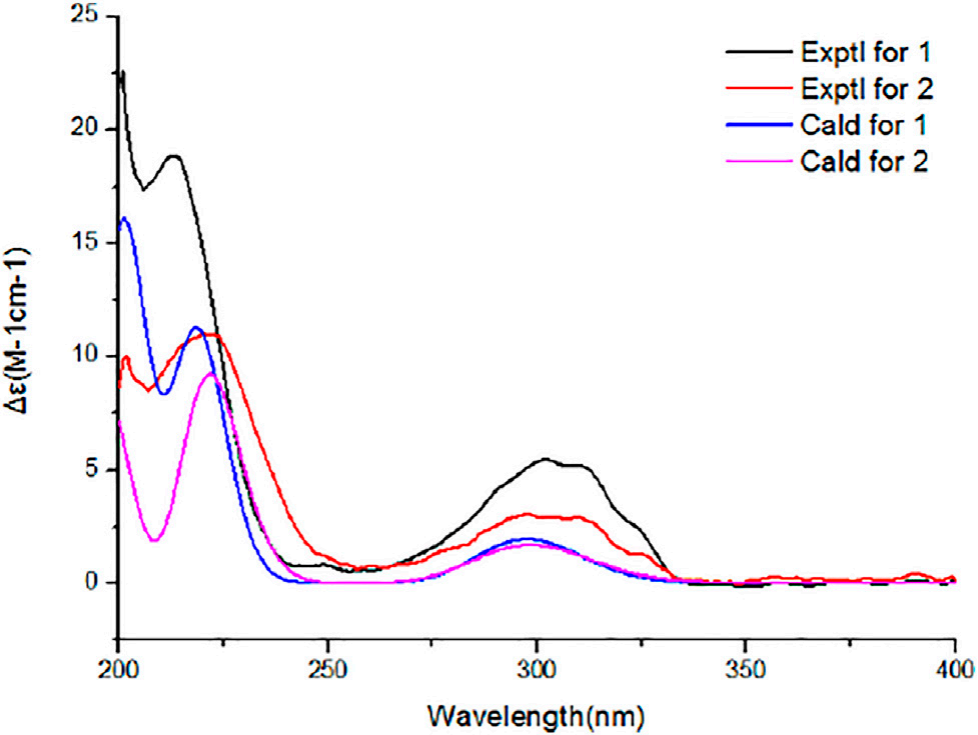
Calculated and experimental electronic circular dichroism spectra of **1** and **2**.

Aiming at determining the absolute configuration of **1**, the ECD calculation was performed at the WB97XD/CC-PVDZ level. The results showed that the calculated ECD curve was in good agreement with the experimental one (Figure 5). Therefore, the absolute configuration of **1** was assigned as 1*R*, 3*S*, 4*R*, 5*S*, 6*S*, 7*S*, 10*S*, 21*R*, 15*R*, 16*S*. The absolute configuration of **2** was determined to be 1*R*, 3*R*, 4*R*, 5*S*, 6*S*, 7*S*, 10*S*, 21*R*, 15*R*, 16*S* by the identical experimental and calculated curves (Figure 5).

Compound **5** was obtained as a yellow solid. The molecular formula was determined to be C_12_H_13_O_5_ based on HRESIMS data (*m/z* 237.07583 [M + H]^+^). The ^1^H NMR spectrum (Table 2) of **5** showed two methyl groups at *δ*_H_ 2.02 (s), 2.44 (s), one methoxyl group at *δ*_H_ 3.96 (s), one olefinic proton at *δ*_H_ 6.41 (s), and one chelated hydroxyl group at *δ*_H_ 12.51 (s). The ^13^C NMR data (Table 2) of **5** highlighted the presence of 12 carbon resonances, including three methyls (one oxygenated), one olefinic carbon, and eight quaternary carbons (one carbonyl carbon and seven olefinic carbons). The ^1^H and ^13^C NMR data of **5** were similar to those of **6**, indicating that **5** was one chromone. The structure of **5** was further established by the HMBC correlations (Figure 2) from H_3_-9 to C-1, C-2, and C-3 and from H_3_-10 to C-3.

Compound **9** was obtained as a white solid. The molecular formula was determined to be C_11_H_9_O_4_ based on HRESIMS data. The ^1^H NMR spectrum (Table 3) of **9** showed one methyl group at *δ*_H_ 2.75 (s), one methoxyl group at *δ*_H_ 3.95 (s), and two aromatic protons at *δ*_H_ 7.02 (d, *J* = 2.2 Hz),6.44 (d, *J* = 2.2 Hz). The ^13^C NMR data (Table 3) of **9** highlighted the presence of 11 carbon resonances, including two methyls, two sp^2^ methines, and seven quaternary carbons. These data suggest **9** to be a benzofuran derivate. The NMR data of **9** were closely similar to penicifuran C (Qi et al., 2013), except for the presence of a methoxyl group, The HMBC correlations (Figure 2) from H_3_-10 to C-7 indicated that the methoxyl group was located at C-7. Thus, the structure of **9** was determined as shown in Figure 1.

Compound **10** was obtained as a white solid. The molecular formula was determined to be C_10_H_13_O_5_ based on HRESIMS data. The ^1^H and ^13^C NMR data (Table 3) of **10** were similar to those of **9**, except that the aldehyde group in **9** was oxidized to the carboxyl group, and there was an absence of the methoxyl group. The deduction was further confirmed by the HMBC correlations (Figure 2) from H_3_-8 to C-2, C-3, and C-9. Therefore, the structure of **10** was established as shown.

Compound **11** had the molecular formula of C_15_H_18_O_5_ by the HRESIMS data. The ^1^H NMR spectrum (Table 4) of **11** showed one chelated hydroxyl group at *δ*_H_ 11.10 (s), one methyl group at *δ*_H_ 0.96 (t, *J* = 6.9 Hz), one methoxyl group at *δ*_H_ 3.87 (s), and three olefinic protons at *δ*_H_ 6.29 (s), 6.33 (d, *J* = 2.2 Hz), and 6.48 (d, *J* = 2.2 Hz). The ^13^C NMR data (Table 4) of **11** revealed the presence of 15 carbon resonances, including two methyls, three methylenes, one sp^3^ and three sp^2^ methines, and six quaternary carbons. These data suggest **11** to be an isocoumarin class. The spin system of H_2_-9/H-10/H_2_-11/H_2_-12/H_3_-13 in the ^1^H-^1^H COSY spectrum (Figure 2) as well as the HMBC correlations (Figure 2) from H_2_-9 to C-3 and C-4 showed that the side chain was substituted at C-3. By comparing the specific rotation of **11** [[α]−21.5 (с 0.06, MeOH)] with (−)-citreoisocoumarin [[α] −29.8 (*с* 0.34, MeOH)] (Mallampudi et al., 2020), the 10*R* configuration at C-10 in **11** was indicated. Thus, the gross structure of **11** was defined as shown.

Compound **12** was isolated as a colorless oil. The molecular formula was determined to be C_15_H_18_O_5_ based on HRESIMS data. The comparison of the ^1^H and ^13^C NMR data (Table 4) with those of **11** revealed that they share the same isocoumarin structure, except that the hydroxyl group was substituted at C-12 in 12. The spin system of H_2_-9/H_2_-10/H_2_-11/H-12/H_2_-13 in the ^1^H-^1^H COSY spectrum (Figure 2), together with the HMBC correlations (Figure 2) from H_2_-9 to C-3 and C-4, further supported this possibility. The 12*S* configuration was confirmed by the positive specific rotation value of **12** [[α] +18.6 (*с* 0.07, MeOH)] when compared with peneciraistin D [[α] +21.1 (*с* 0.14, MeOH)] (Ma et al., 2012).

Compound **13** was isolated as a colorless oil with the molecular formula of C_15_H_14_O_6_ based on HRESIMS data. Upon comparing the ^1^H and ^13^C NMR data (Table 4) between **11** and **13**, it was suggested that **13** also possessed the isocoumarin framework. The spin system of H-9/H-10 observed in the ^1^H-^1^H COSY spectrum (Figure 2) and the HMBC correlations (Figure 2) from H-9 to C-3 and C-4 from H-10 to C-11 made it possible to obtain the gross structure. Additionally, an ethyl group was linked with C-11 by the HMBC correlations from H-12 to C-11. The 9*E* configuration of the double bond was determined by the large coupling constant *J*_H-9, H-10_ = 15.5 Hz. Thus, the structure of **13** was confirmed as shown in Figure 1.

Five known analogues were characterized as equisetin (**3**) (Zhao et al., 2019), epi-equisetin (**4**) (Zhao et al., 2019), takanechromone B (**6**) (Qader et al., 2021), altechromone A (**7**) (Tanaka et al., 2009), 4H-1-benzopyran-4-one- 2,3-dihydro-5-hydroxy-8-(hydroxylmethyl)-2-methyl (**8**) (Sousa et al., 2016), and aspergisocoumrin A (**14**) (Wu et al., 2019) through a comparison of the spectroscopic data with the literature.

All compounds were evaluated for their cytotoxicity against the A549 (lung carcinoma), HELA (cervical carcinoma), KYSE150 (esophageal squamous carcinoma), PC-3 (pancreatic carcinoma), and MDA-MB-435 (breast carcinoma) human cancer cell lines (Table 5). As a result, compounds **1** and **2** showed selective cytotoxicity against A549 cell line with IC_50_ values of 8.7 and 4.3 μM, respectively. Compound **8** showed potent cytotoxicity against A549 and MDA-MB-435 cell lines with IC_50_ values of 5.6 and 3.8 μM, respectively. Compound **14** exhibited significant cytotoxicity against A549 and MDA-MB-435 cell lines with IC_50_ values of 6.2 and 2.8 μM, respectively, while the other compounds exhibited non-significant activity against the five cancer cell lines at the concentration of 50 μM.

**TABLE 5:** Cytotoxicity of compounds **1–4**, **8** and **13** (IC_50_ ± SD, μM).

Compound	A549	HELA	KYSE150	PC-3	MDA-MB-435
**1**	8.7 ± 0.6	39.2 ± 0.7	36.3 ± 0.5	>50	>50
**2**	4.3 ± 0.2	>50	>50	>50	>50
**4**	15.3 ± 0.6	>50	>50	>50	>50
**8**	5.6 ± 1.3	>50	>50	>50	3.8 ± 0.3
**13**	>50	>50	>50	>50	30.5 ± 0.1
**14**	6.2 ± 0.2	—	—	—	2.8 ± 0.8
DDP^a^	25.9 ± 0.8	10.0 ± 0.1	72.6 ± 4.3	41.6 ± 0.9	9.6 ± 0.9

a Positive control. “—” not tested.The IC_50_ values were expressed as means ± SD (*n* = 3) from three independent experiments.

## Conclusion

In summary, two new 3-decalinoyltetramic acid (3DTA) derivatives, fusarisetins E (**1**) and F (**2**), with a peroxide bridge, were isolated from mangrove endophytic fungus *Fusarium* sp. 2ST2. The 3DTA derivatives showed various bioactivities, such as antimicrobial, anticancer, larvicidal, cytotoxic, and antiviral (Fan et al., 2020). The structure of fusarisetins E (**1**) and F (**2**) was similar to that of fusarisetin A, which was first isolated from the soil fungus *Fusarium* sp. FN080326 with inhibitory activity to acinar morphogenesis (Jang et al., 2011), while fusarisetin E (**1**) was identified as peroxyfusarisetin (Yin et al., 2012), a synthetic intermediate by mixture. Here fusarisetin E (**1**) was reported first as an optically pure new natural product with 1D and 2D NMR data (Supplementary Figures S1–S8). Moreover, natural peroxide compounds that usually have unique pharmacological activities, such as artemisinin with antimalarial activity (Zhao et al., 2018; Pandey et al., 1999), talaperoxides A-D with cytotoxicity (Li et al., 2011), phaeocaulisin M with anti-inflammatory activity (Ma et al., 2015), 1*α*,8*α*-epidioxy-4*α*-hydroxy-5*α*H-guai-7(11),9-dien-12,8-olide with antiviral activity (Dong et al., 2013), and plakinic acid M with antifungal activity (Matthew et al., 2016), were reported. Compounds **1** and **2** had selective cytotoxicity against A549 cell line with IC_50_ values of 8.7 and 4.38 μM, respectively. The cytotoxicity of fusarisetins was reported for the first time.

## Data Availability

The original contributions presented in the study are included in the article/Supplementary Material, further inquiries can be directed to the corresponding authors.
