# Supplementary material for: Metabolites With Cytotoxic Activities From the Mangrove Endophytic Fungus Fusarium sp. 2ST2
Source: Front Chem. 2022 Feb 15;10:842405. doi: 10.3389/fchem.2022.842405 (PMC8885587; doi:10.3389/fchem.2022.842405)
Supplement: Supplementary file 1 [file DataSheet1.docx]

Supplementary Material

Metabolites with Cytotoxic Activities from the Mangrove Endophytic Fungus *Fusarium* sp. 2ST2

Yan Chen^1,2^, Guisheng Wang^1^, Yilin Yuan^1^, Ge Zou^2^, Wencong Yang^2^, Qi Tan^2^, Wenyi Kang ^1^* and Zhigang She ^2^*

^1^ National R & D Center for Edible Fungus Processing Technology, Henan University, Kaifeng 475004, China; chenyan27@mail2.sysu.edu.cn (Y.C.); 104754201481@henu.edu.cn (G.W.); yilin@henu.edu.cn (Y.Y.);

^2^ School of Chemistry, Sun Yat-Sen University, Guangzhou 510275, China; zoug5@mail2.sysu.edu.cn (G.Z.); yangwc6@mail2.sysu.edu.cn (W.Y.); tanq27@mail2.sysu.edu.cn (Q.T.);

***** Correspondence: kangwenyi@henu.edu.cn (W.K.); cesshzhg@mail.sysu.edu.cn (Z.S.).

**Supporting Information Contents:**

**Figure S1.** ^1^H NMR spectrum of compound **1** (500 MHz, CD_3_OD-*d*_4_).

**Figure S2.** ^1^H NMR spectrum of compound **1** (500 MHz, DMSO-*d*_6_).

**Figure S3.** ^13^C NMR spectrum of compound **1** (125 MHz, CD_3_OD-*d*_4_).

**Figure S4.** HSQC spectrum of compound **1**.

**Figure S5.** ^1^H-^1^H COSY spectrum of compound **1**.

**Figure S6.** HMBC spectrum of compound **1**.

**Figure S7.** NOESY spectrum of compound **1**.

**Figure S8.** NOESY spectrum of compound **1** (DMSO-*d*_6_).

**Figure S9.** HRESIMS spectrum of compound **1**.

**Figure S10.** ^1^H NMR spectrum of compound **2** (500 MHz, CD_3_OD-*d*_4_).

**Figure S11.** ^1^H NMR spectrum of compound **2** (500 MHz, DMSO-*d*_6_).

**Figure S12.** ^13^C NMR spectrum of compound **2** (125 MHz, CD_3_OD-*d*_4_).

**Figure S13.** HSQC spectrum of compound **2**.

**Figure S14.** ^1^H-^1^H COSY spectrum of compound **2**.

**Figure S15.** HMBC spectrum of compound **2**.

**Figure S16.** NOESY spectrum of compound **2**.

**Figure S17.** NOESY spectrum of compound **2** (DMSO-*d*_6_).

**Figure S18.** HRESIMS spectrum of compound **2**.

**Figure S19.** ^1^H NMR spectrum of compound **3** (500 MHz, CDCl_3_).

**Figure S20.** ^13^C NMR spectrum of compound **3** (125 MHz, CDCl_3_).

**Figure S21.** ^1^H NMR spectrum of compound **4** (500 MHz, CDCl_3_).

**Figure S22.** ^13^C NMR spectrum of compound **4** (125 MHz, CDCl_3_).

**Figure S23.** ^1^H NMR spectrum of compound **5** (500 MHz, CDCl_3_).

**Figure S24.** ^13^C NMR spectrum of compound **5** (125 MHz, CDCl_3_).

**Figure S25.** HSQC spectrum of compound **5**.

**Figure S26.** HMBC spectrum of compound **5**.

**Figure S27.** HRESIMS spectrum of compound **5**.

**Figure S28.** ^1^H NMR spectrum of compound **9** (500 MHz, CD_3_OD-*d*_4_)

**Figure S29.** ^13^C NMR spectrum of compound **9** (500 MHz, CD_3_OD-*d*_4_)

**Figure S30**. HSQC spectrum of compound **9**

**Figure S31.** HMBC spectrum of compound **9**

**Figure S32.** HRESIMS spectrum of compound **9**

**Figure S33.** ^1^H NMR spectrum of compound **10** (500 MHz, CD_3_OD-*d*_4_)

**Figure S34.** ^13^C NMR spectrum of compound**10** (500 MHz, CD_3_OD-*d*_4_)

**Figure S35.** HMBC spectrum of compound **10**.

**Figure S36.** HRESIMS spectrum of compound **10**

**Figure S37.** ^1^H NMR spectrum of compound **11** (500 MHz, CDCl_3_)

**Figure S38.** ^13^C NMR spectrum of compound **11** (500 MHz, CDCl_3_)

**Figure S39.** HSQC spectrum of compound **11**

**Figure S40.** ^1^H-^1^H COSY spectrum of compound **11**

**Figure S41.** HMBC spectrum of compound **11**.

**Figure S42.** HRESIMS spectrum of compound **11**

**Figure S43.** ^1^H NMR spectrum of compound **12** (500 MHz, CDCl_3_)

**Figure S44.** ^13^C NMR spectrum of compound **12** (500 MHz, CDCl_3_)

**Figure S45.** HSQC spectrum of compound **12**

**Figure S46.** ^1^H-^1^H COSY spectrum of compound **12**

**Figure S47.** HMBC spectrum of compound **12**

**Figure S48.** HRESIMS spectrum of compound **12**

**Figure S49.** ^1^H NMR spectrum of compound **13** (500 MHz, CDCl_3_)

**Figure S50.** ^13^C NMR spectrum of compound **13** (500 MHz, CDCl_3_)

**Figure S51**. HSQC spectrum of compound **13**

**Figure S52**. ^1^H-^1^H COSY spectrum of compound **13**

**Figure S53.** HMBC spectrum of compound **13**.

**Figure S54.** NOESY spectrum of compound **13**

**Figure S55.** HRESIMS spectrum of compound **13**

**Table S1.** Energy analysis and the Boltzmann Distribution for fusarisetin E (**1**).

**Table S2.** The optimized lowest energy conformers for fusarisetin E (**1**).

**Table S3.** The DP4+ evaluation of fusarisetin E (**1**).

**Table S4.** Energy analysis and the Boltzmann Distribution for fusarisetin F (**2**).

**Table S5.** The optimized lowest energy conformers for fusarisetin F (**2**).

**Table S6.** The DP4+ evaluation of fusarisetin F (**2**).

**Table S7.** Cartesian coordinates of fusarisetin E (**1**).


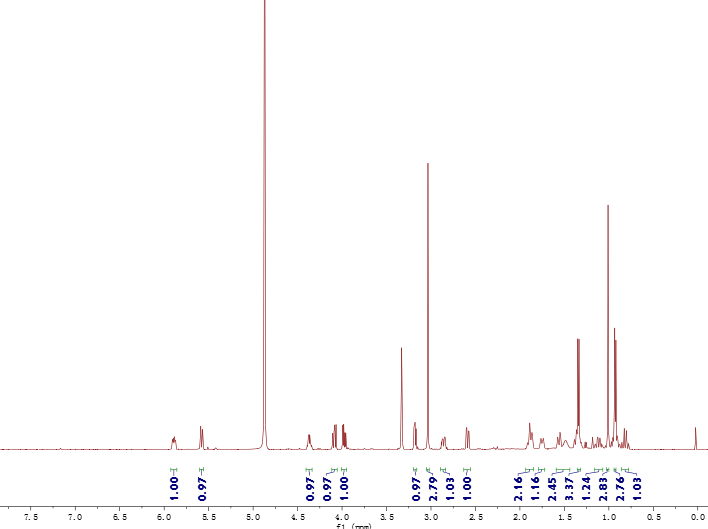


**Figure. S1** ^1^H NMR spectrum of compound **1** (500 MHz, CD_3_OD).


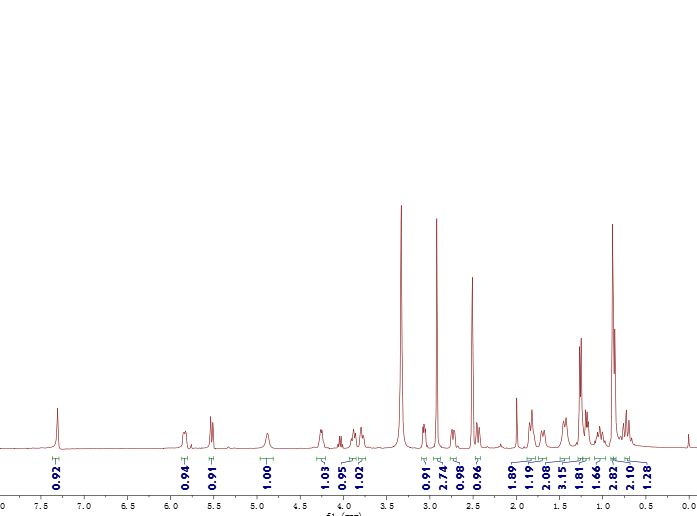


**Figure. S2** ^1^H NMR spectrum of compound **1** (500 MHz, DMSO-*d*_6_)


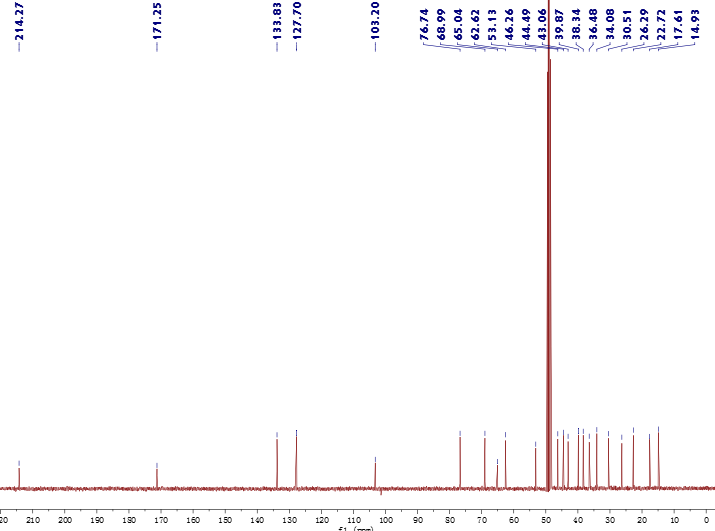


**Figure. S3** ^13^C NMR spectrum of compound **1** (125 MHz, CD_3_OD).


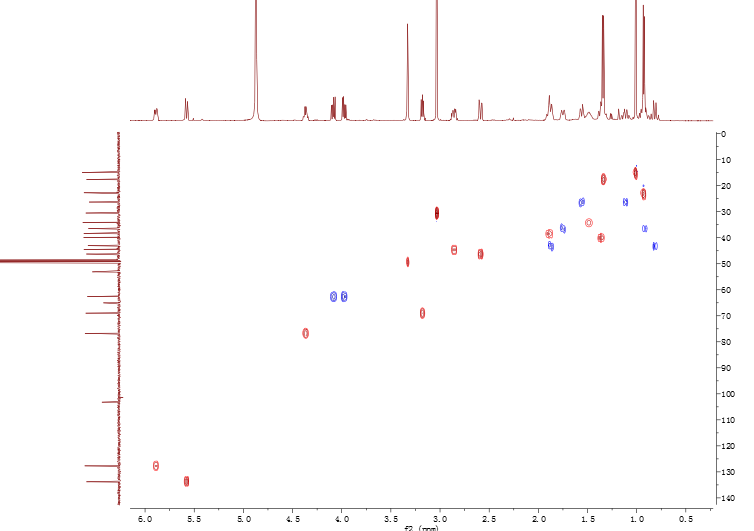


**Figure. S4** HSQC spectrum of compound **1**.


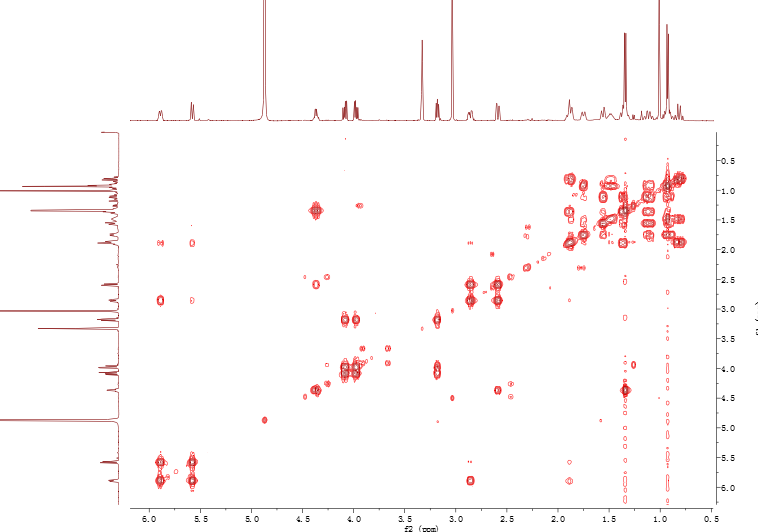


**Figure. S5** ^1^H-^1^H COSY spectrum of compound **1**.


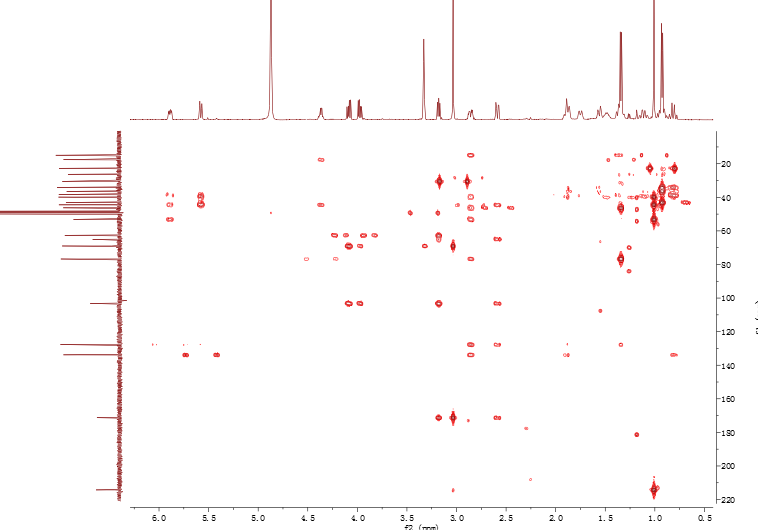


**Figure. S6** HMBC spectrum of compound **1**.


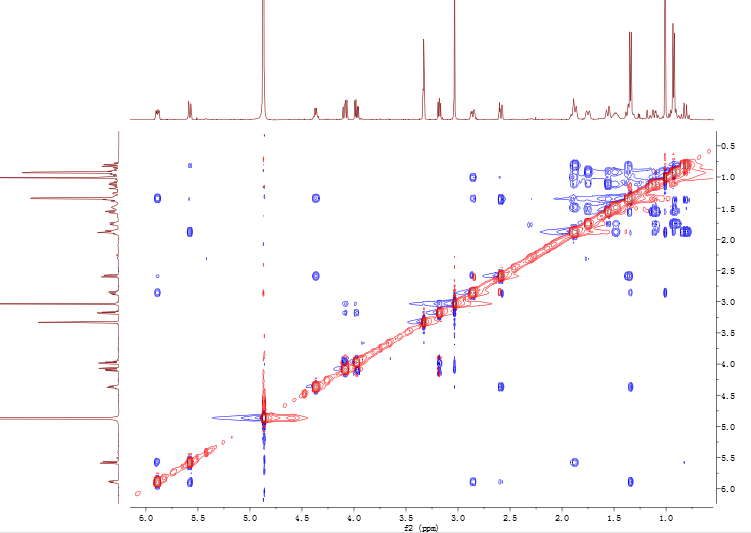


**Figure. S7** NOESY spectrum of compound **1**.


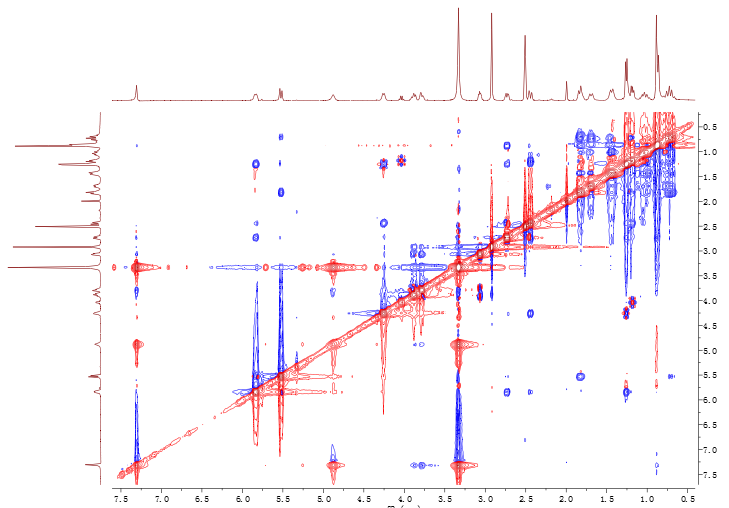


**Figure. S8** NOESY spectrum of compound **1** (DMSO-*d*_6_).

**Figure. S9** HRESIMS spectrum of compound **1**.


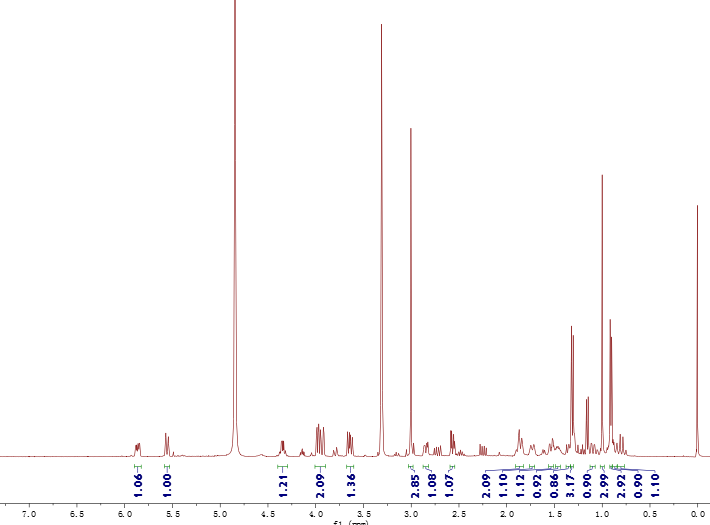


**Figure. S10** ^1^H NMR spectrum of compound **2** (500 MHz, CD_3_OD).


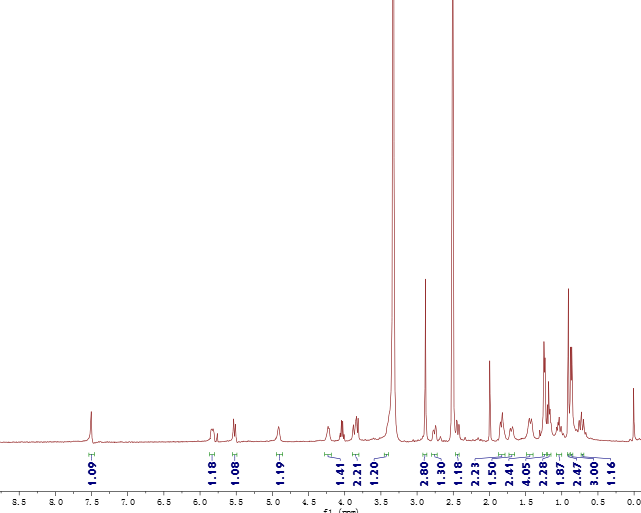


**Figure. S11** ^1^H NMR spectrum of compound **2** (500 MHz, DMSO-*d*_6_).


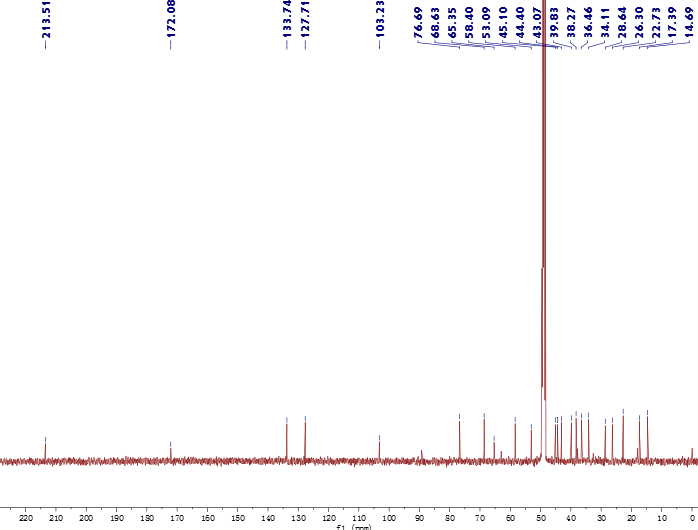


**Figure. S12** ^13^C NMR spectrum of compound **2** (125 MHz, CD_3_OD)


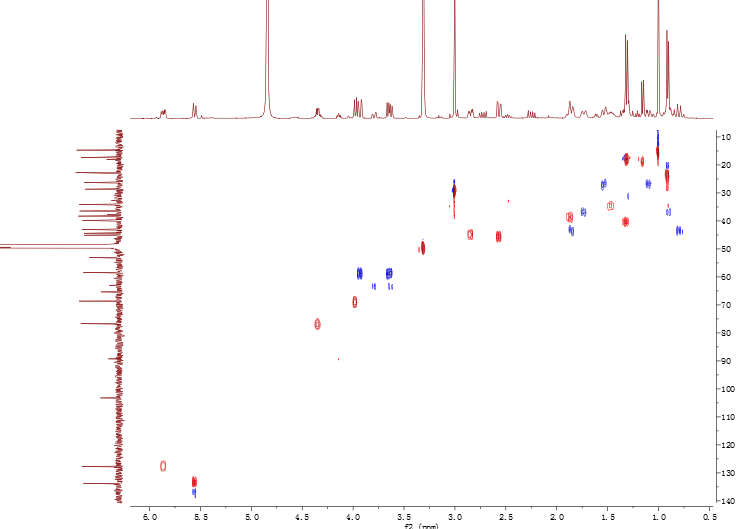


**Figure. S13** HSQC spectrum of compound **2**.


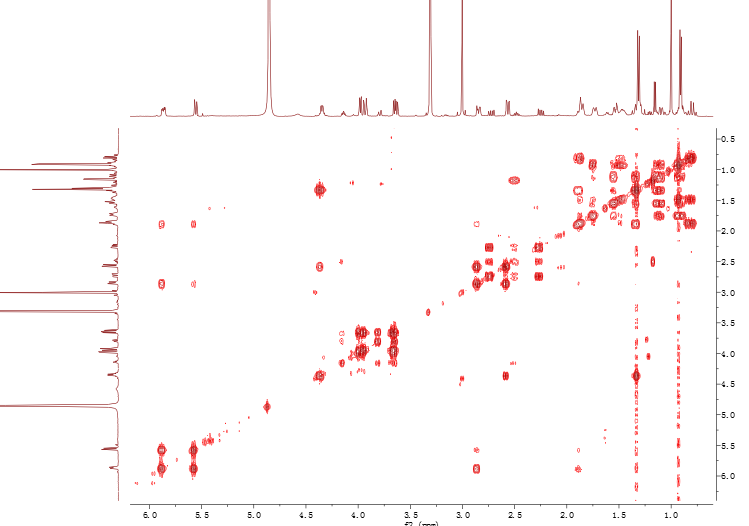


**Figure. S14** ^1^H-^1^H COSY spectrum of compound **2**.


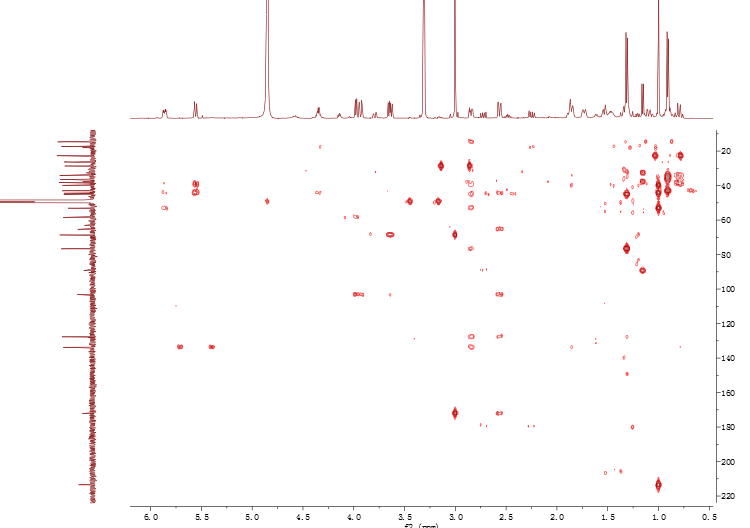


**Figure. S15** HMBC spectrum of compound **2**.


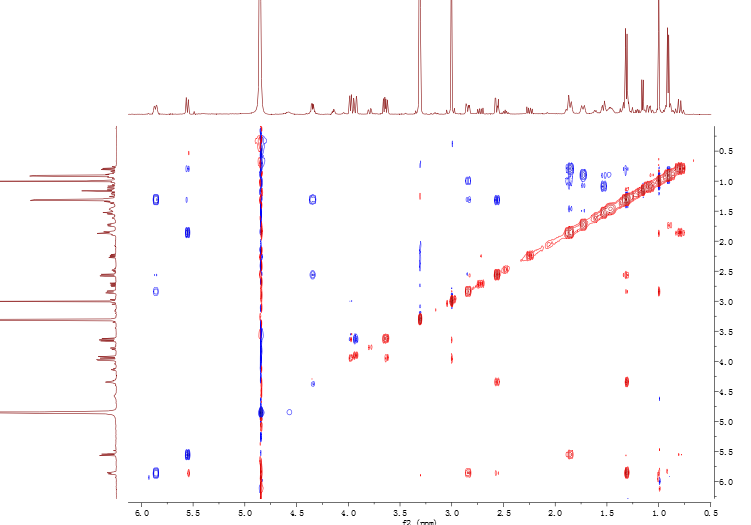


**Figure. S16** NOESY spectrum of compound **2**.


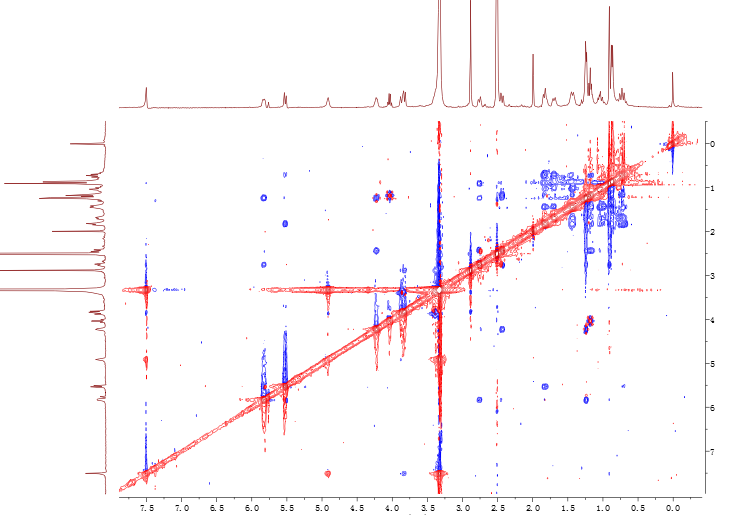


**Figure. S17** NOESY spectrum of compound **2** (DMSO-*d*_6_).

**Figure. S18** HRESIMS spectrum of compound **2**.


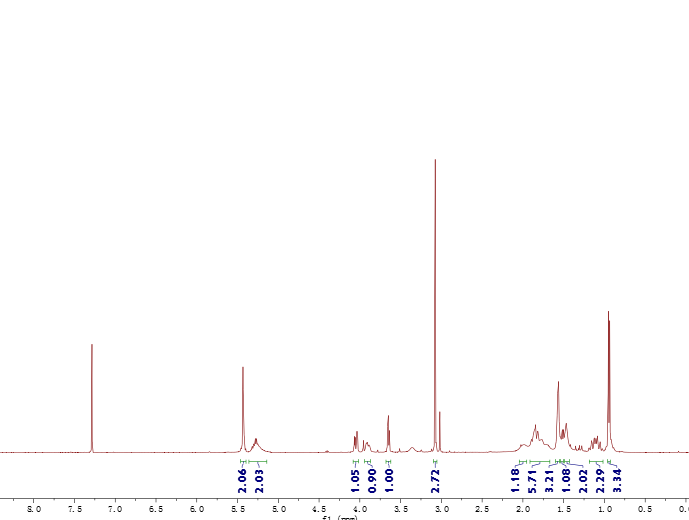


**Figure. S19** ^1^H NMR spectrum of compound **3** (500 MHz, CDCl_3_).


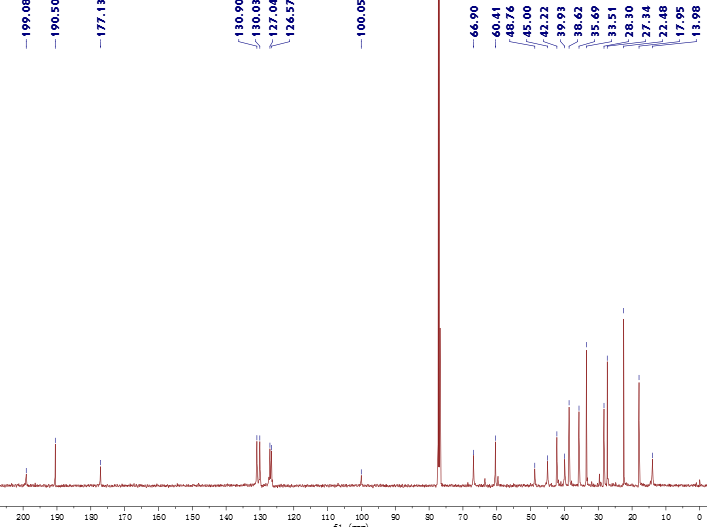


**Figure. S20** ^13^C NMR spectrum of compound **3** (125 MHz, CDCl_3_).


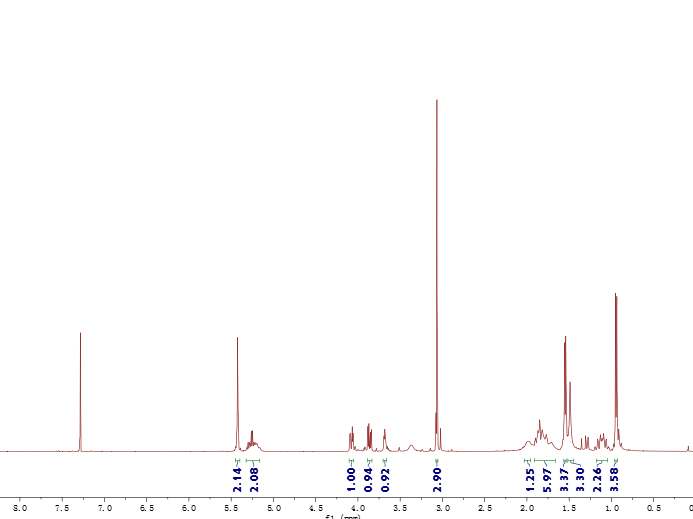


**Figure. S21** ^1^H NMR spectrum of compound **4** (500 MHz, CDCl_3_).


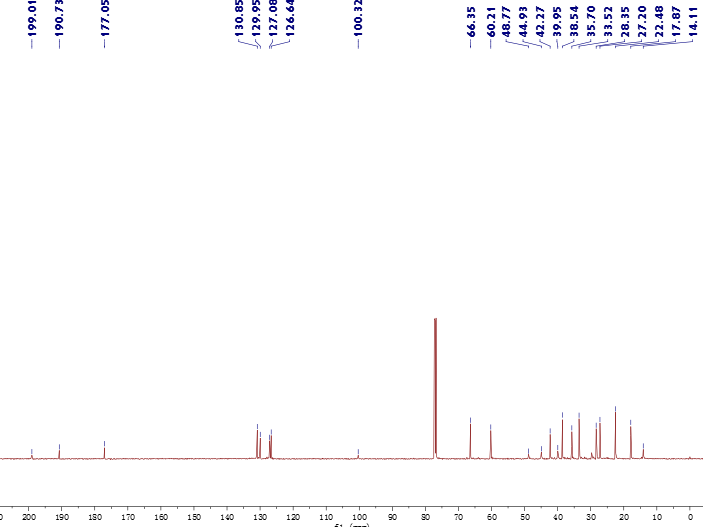


**Figure. S22** ^13^C NMR spectrum of compound **4** (125 MHz, CDCl_3_).


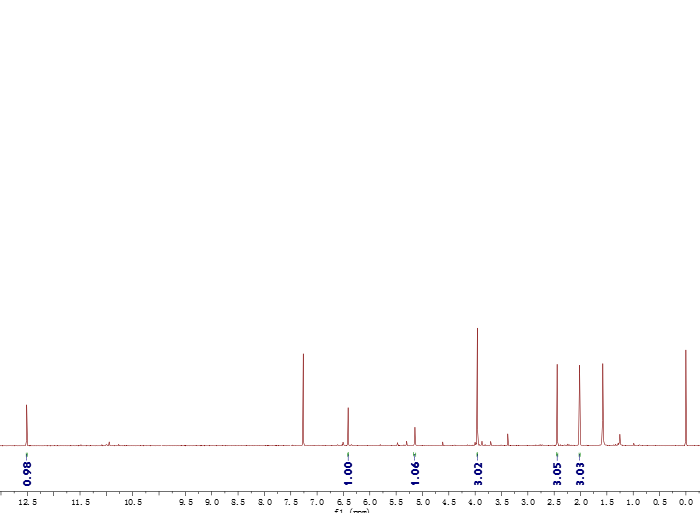


Figure S23. ^1^H NMR spectrum of compound **5** (500 MHz, CDCl_3_)


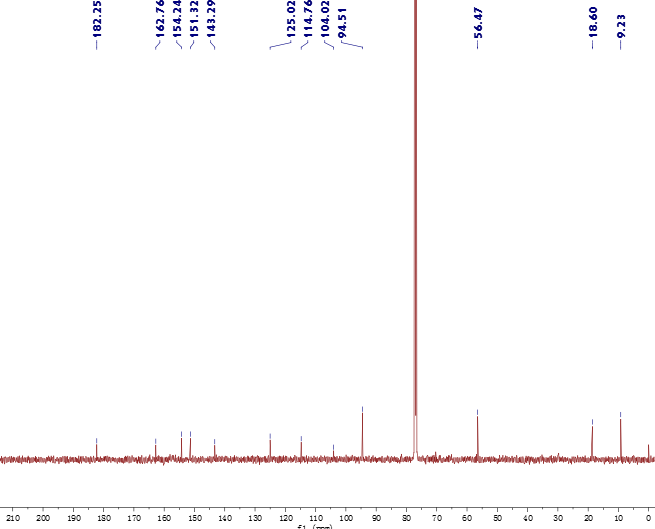


Figure S24. ^13^C NMR spectrum of compound **5** (500 MHz, CDCl_3_)


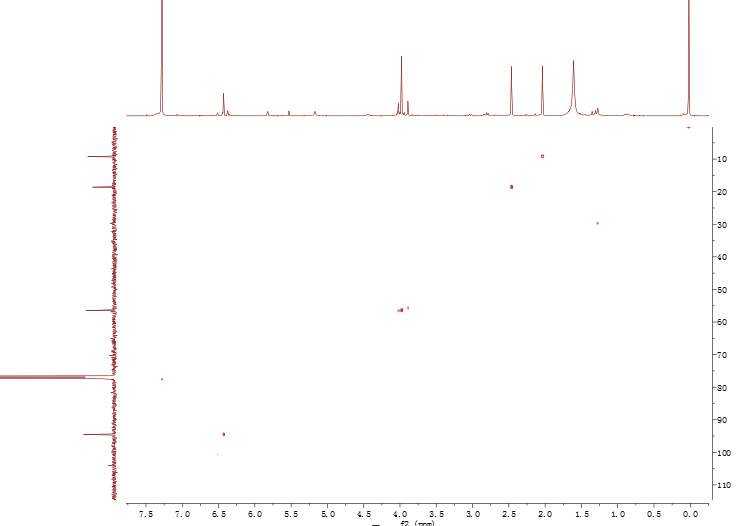


**Figure. S25** HSQC spectrum of compound **5**.


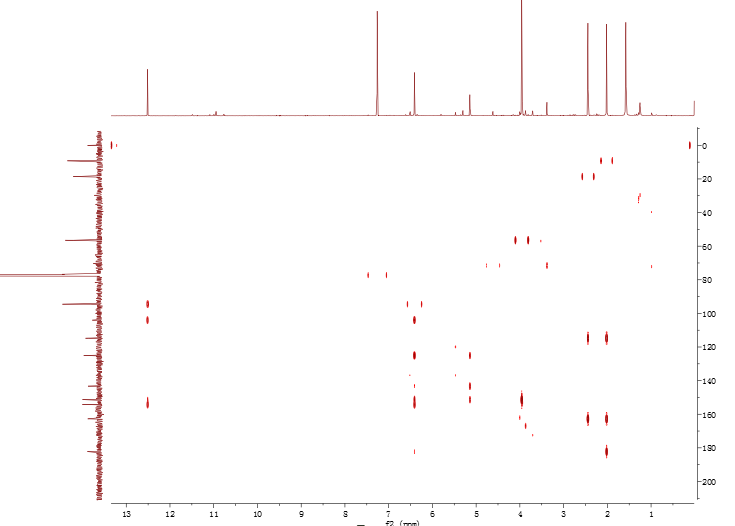


Figure S26. HMBC spectrum of compound **5**.

Figure S27. HRESIMS spectrum of compound **5**


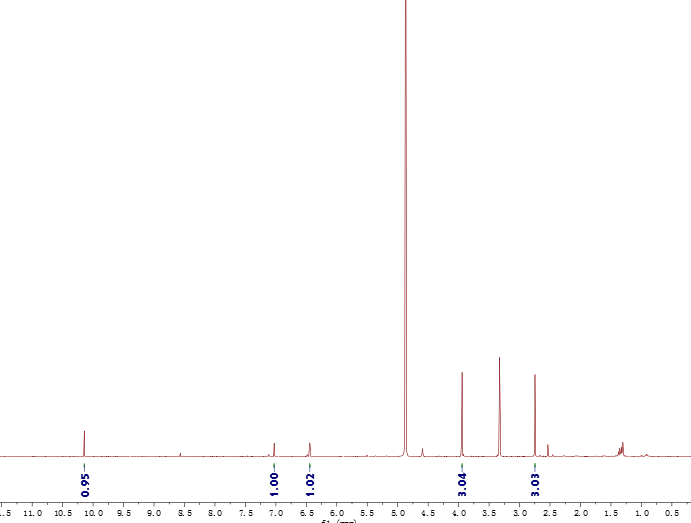


Figure S28. ^1^H NMR spectrum of compound **9** (500 MHz, CD_3_OD-*d*_4_)


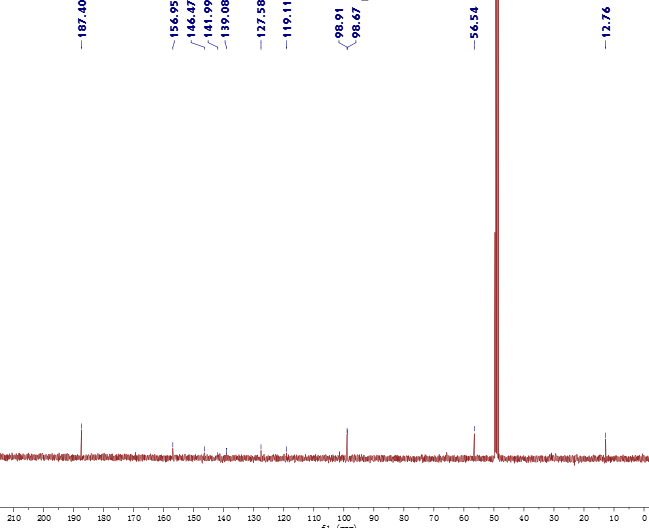


Figure S29. ^13^C NMR spectrum of compound **9** (500 MHz, CD_3_OD-*d*_4_)


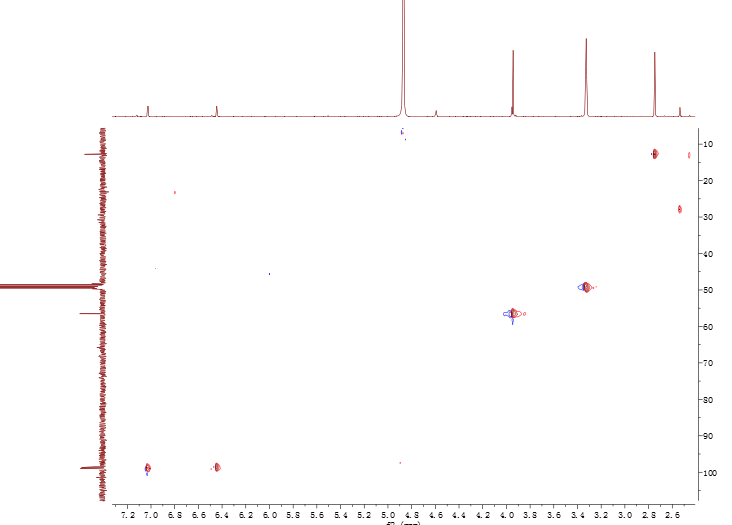


Figure S30. HSQC spectrum of compound **9**


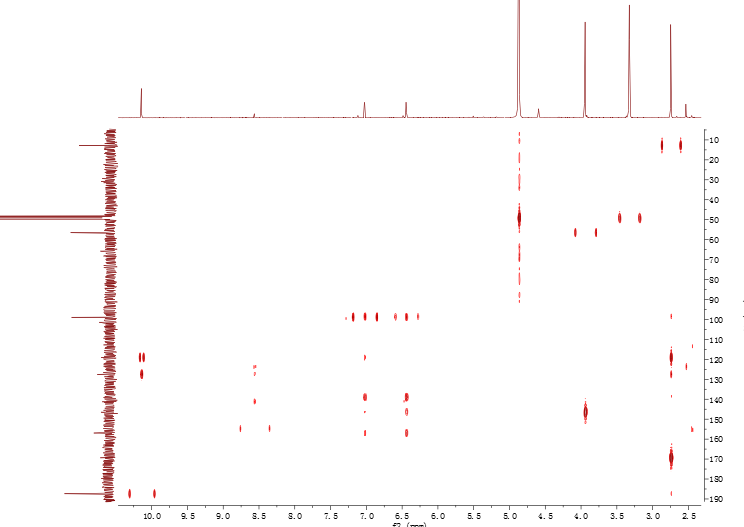


Figure S31. HMBC spectrum of compound **9**

Figure S32. HRESIMS spectrum of compound **9**


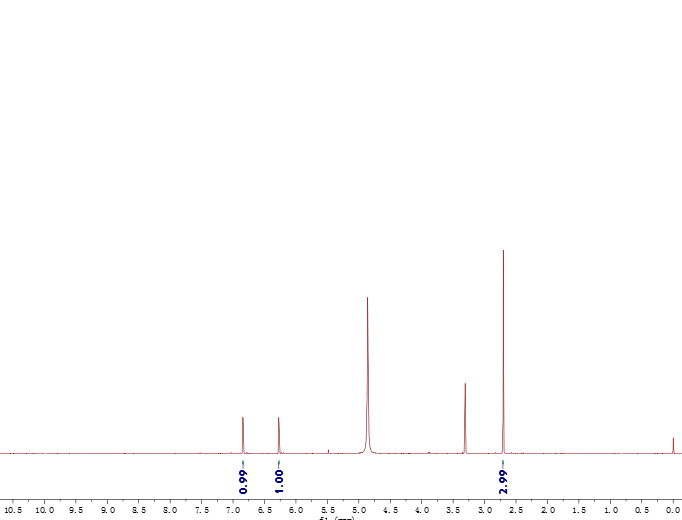


Figure S33. ^1^H NMR spectrum of compound **10** (500 MHz, CD_3_OD-*d*_4_)


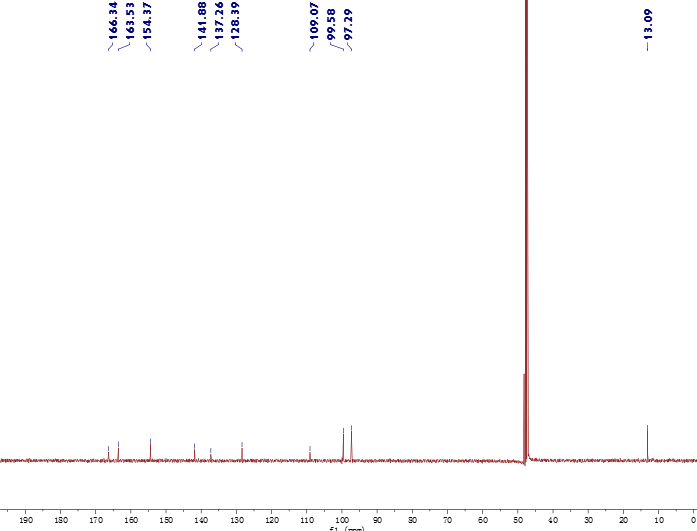


Figure S34. ^13^C NMR spectrum of compound**10** (500 MHz, CD_3_OD-*d*_4_)


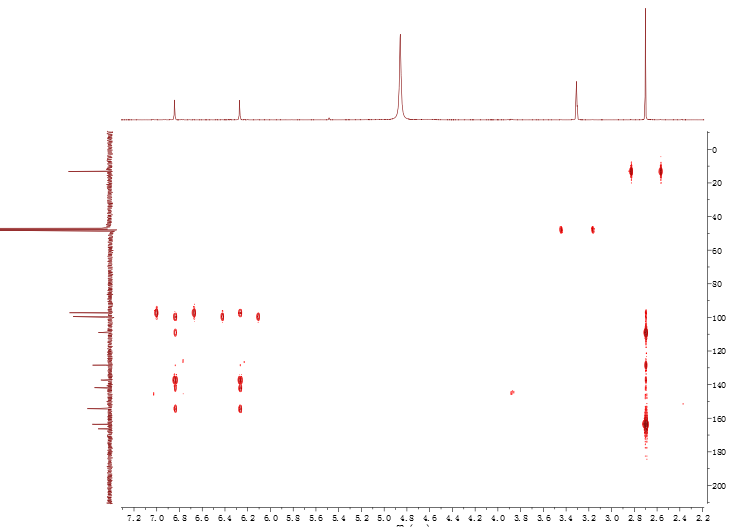


Figure S35. HMBC spectrum of compound **10**.

Figure S36. HRESIMS spectrum of compound **10**


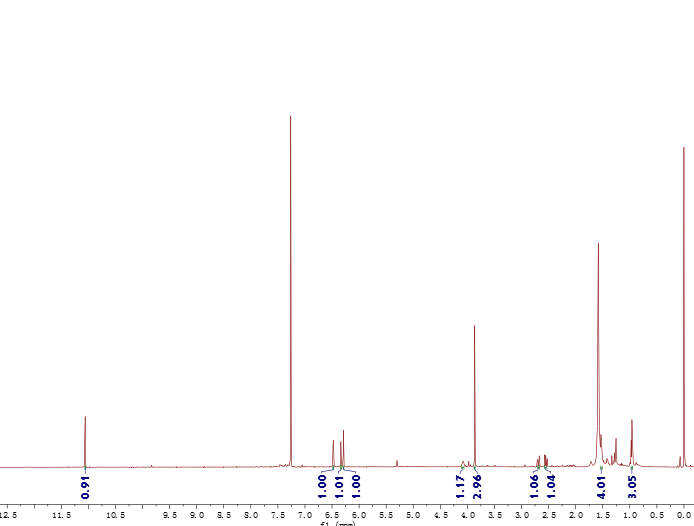


Figure S37. ^1^H NMR spectrum of compound **11** (500 MHz, CDCl_3_)


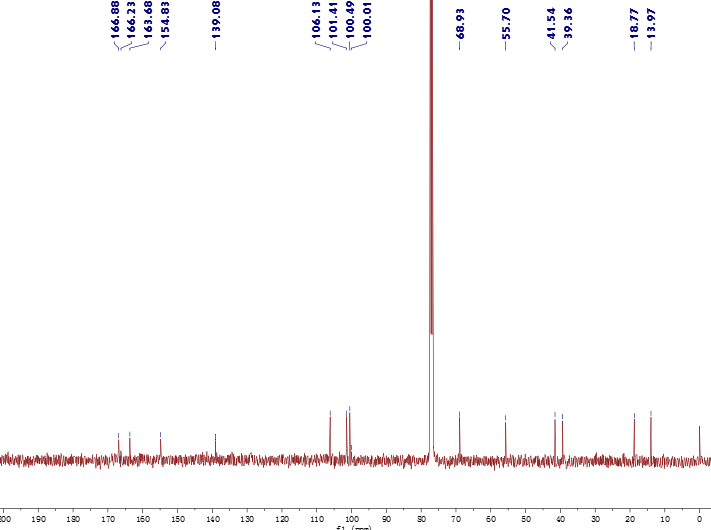


Figure S38. ^13^C NMR spectrum of compound **11** (500 MHz, CDCl_3_)


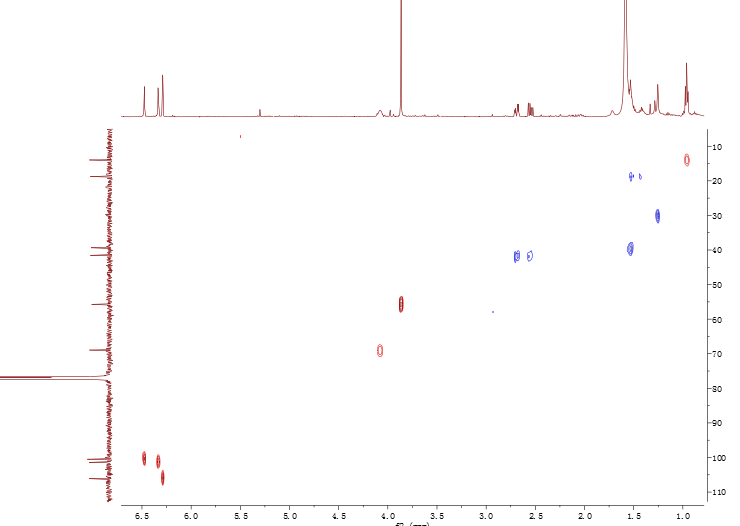


Figure S39. HSQC spectrum of compound **11**


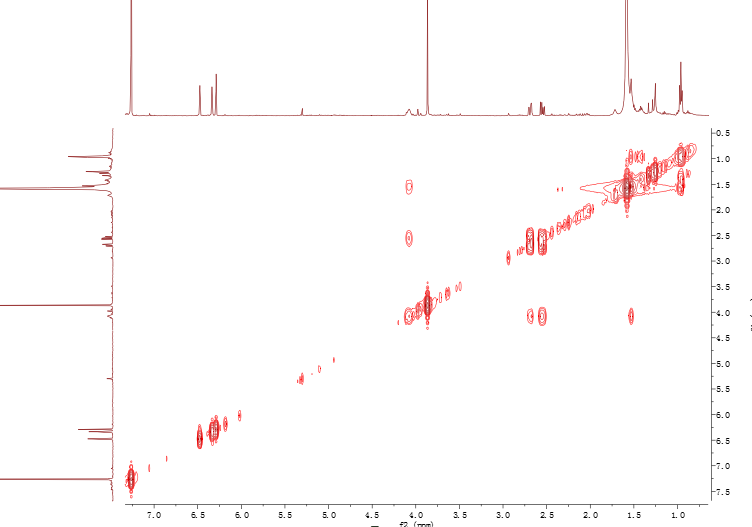


Figure S40. ^1^H-^1^H COSY spectrum of compound **11**


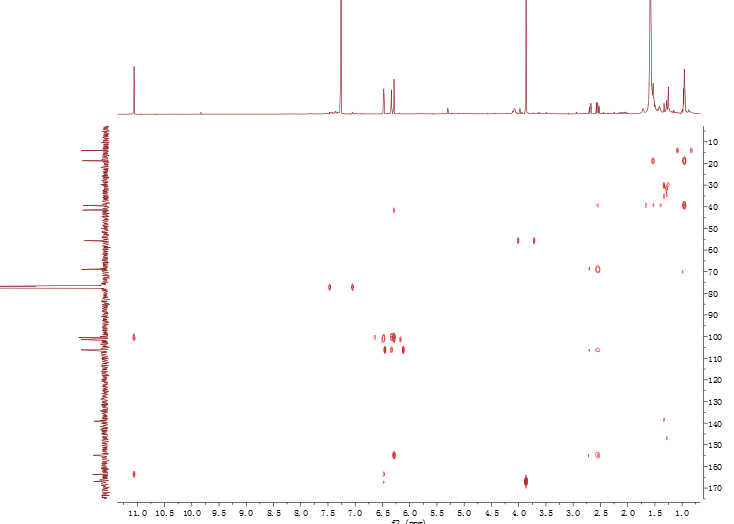


Figure S41. HMBC spectrum of compound **11**.

Figure S42. HRESIMS spectrum of compound **11**


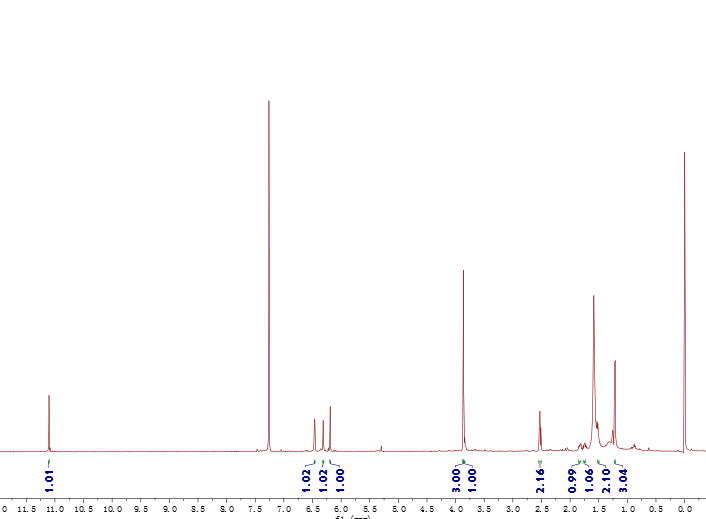


Figure S43. ^1^H NMR spectrum of compound **12** (500 MHz, CDCl_3_)


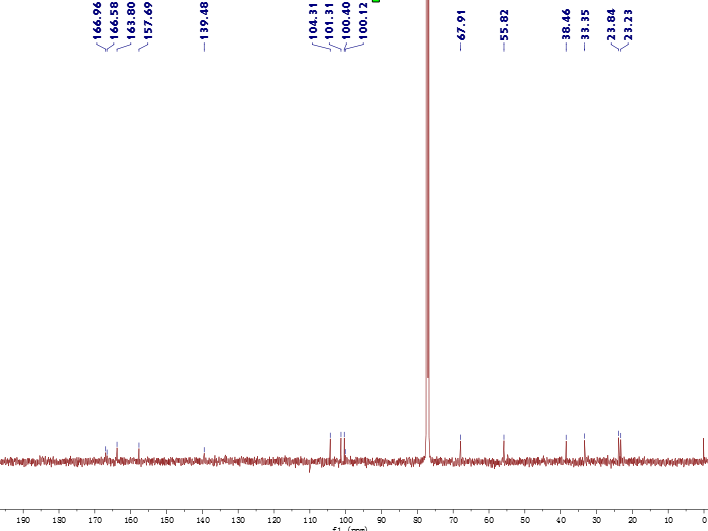


Figure S44. ^13^C NMR spectrum of compound **12** (500 MHz, CDCl_3_)


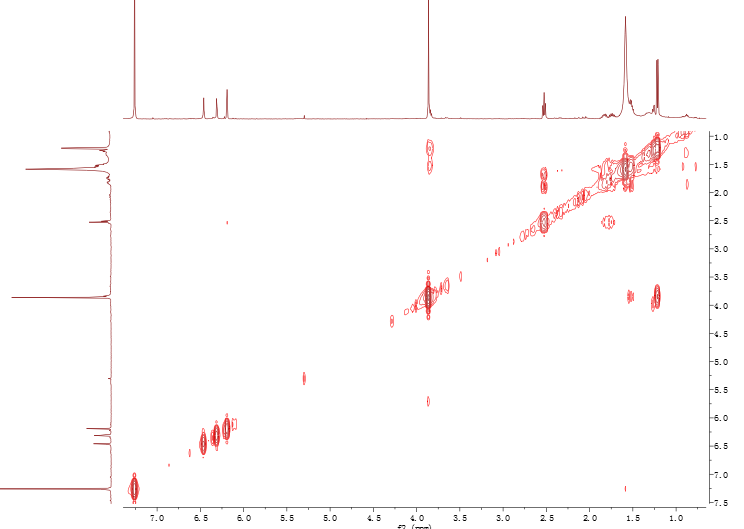


Figure S45. HSQC spectrum of compound **12**


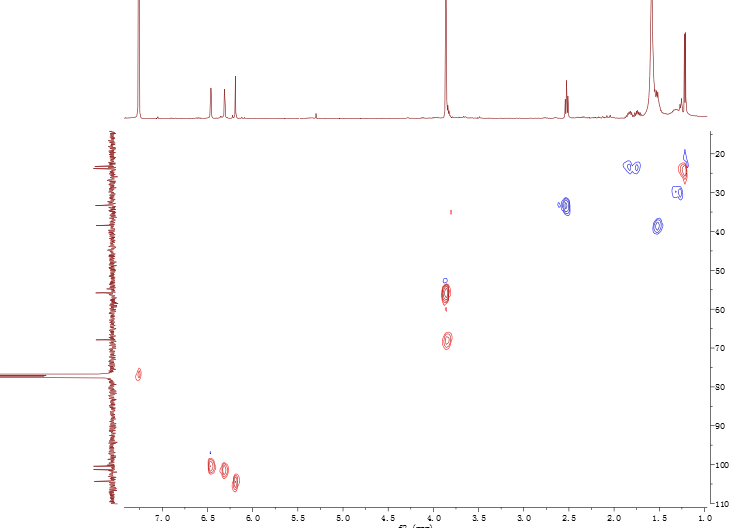


Figure S46. ^1^H-^1^H COSY spectrum of compound **12**


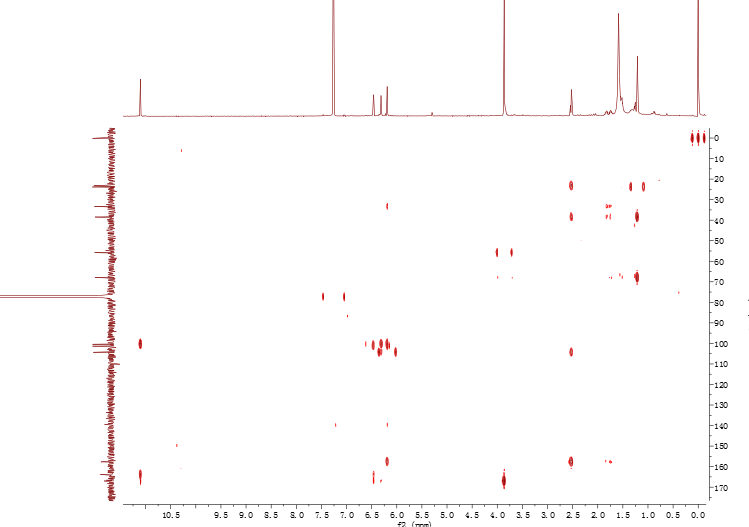


Figure S47. HMBC spectrum of compound **12**

Figure S48. HRESIMS spectrum of compound **12**


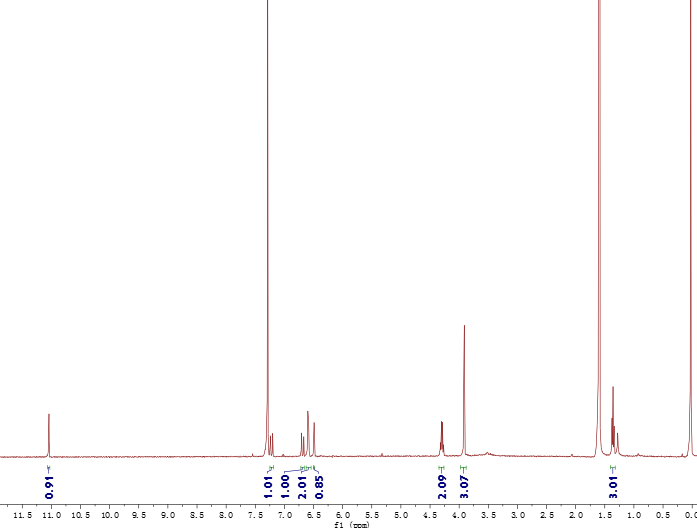


Figure S49. ^1^H NMR spectrum of compound **13** (500 MHz, CDCl_3_)


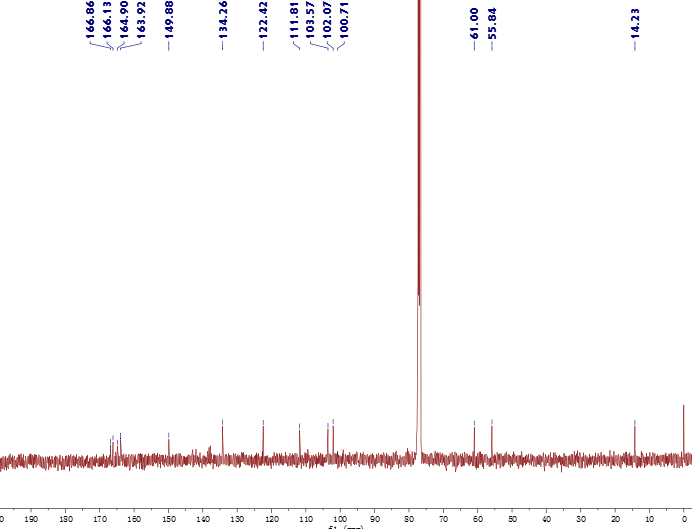


Figure S50. ^13^C NMR spectrum of compound **13** (500 MHz, CDCl_3_)


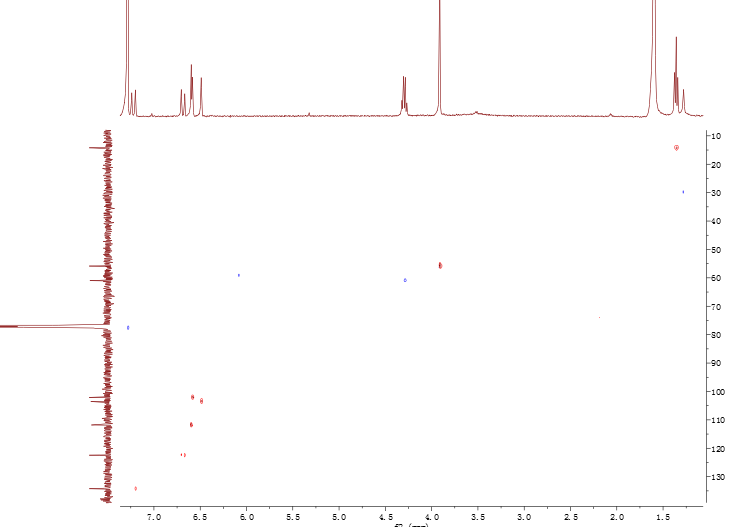


Figure S51. HSQC spectrum of compound **13**


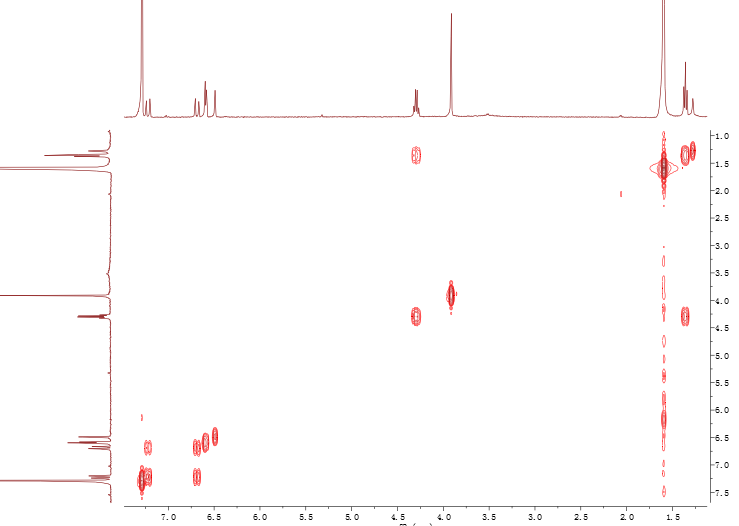


Figure S52. ^1^H-^1^H COSY spectrum of compound **13**


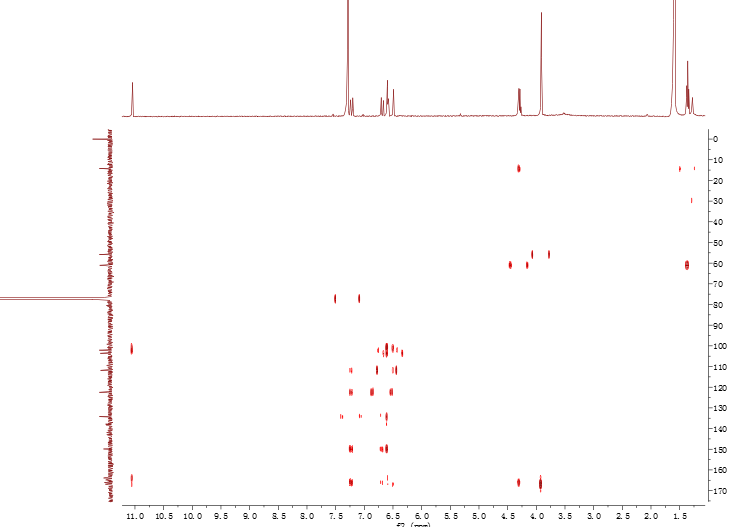


Figure S53. HMBC spectrum of compound **13**.


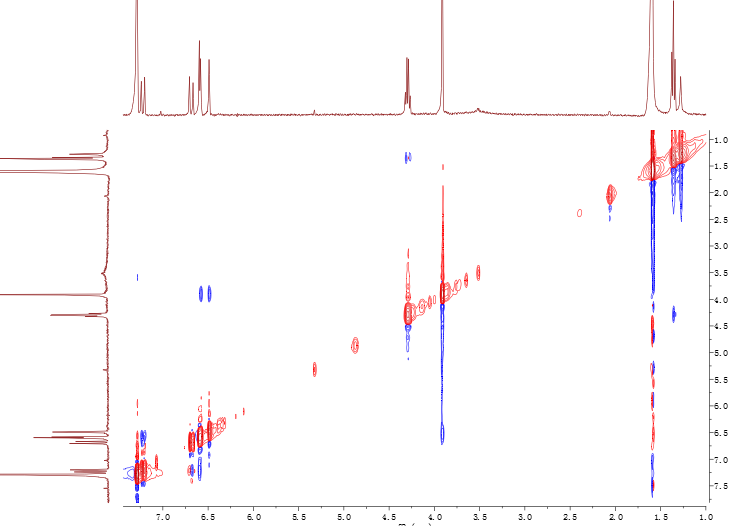


Figure S54. NOESY spectrum of compound **13**.

Figure S55. HRESIMS spectrum of compound **13**

Table S1. Energy analysis and the Boltzmann Distribution for fusarisetin E (**1**).

| Conf. | G (Hartree) | ΔG (KJ/mol) | Boltzmann Distribution |
| --- | --- | --- | --- |
| 1a | | | |
| 1a1 | -1363.13282990 | 3.413615776 | 0.0031 |
| 1a2 | -1363.13328920 | 3.125403694 | 0.0051 |
| 1a3 | -1363.13826990 | 0 | 0.9918 |
| 1b | | | |
| 1b1 | -1363.10936980 | 0.498237303 | 0.3012 |
| 1b2 | -1363.11016380 | 0 | 0.6988 |

Table S2. The optimized lowest energy conformers for fusarisetin E (**1**).


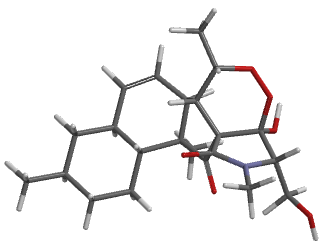

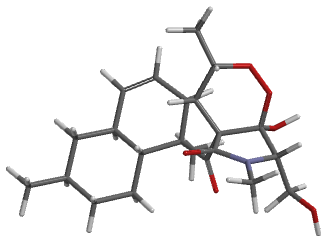

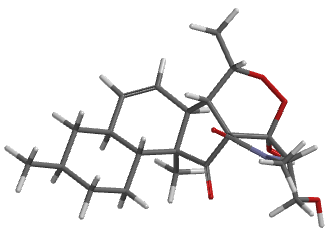


1a1 1a2 1a3


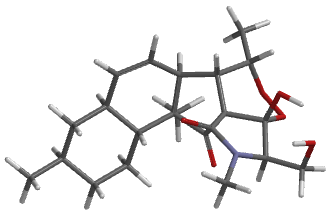

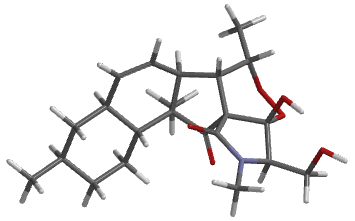


1b1 1b2

Table S3. The DP4+ evaluation of fusarisetin E (**1**).


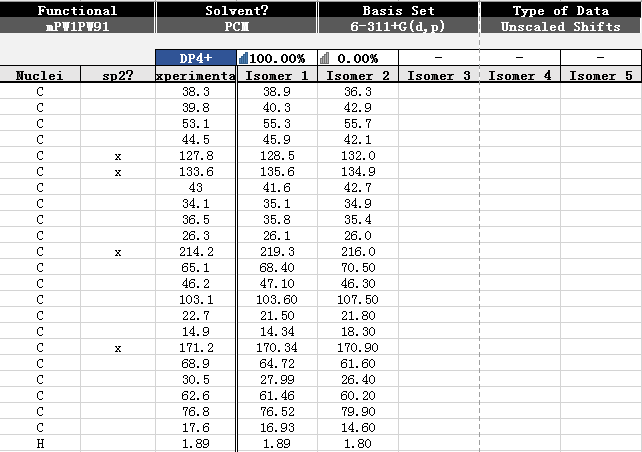

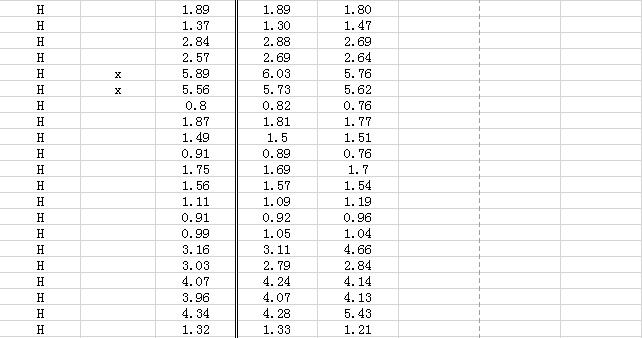


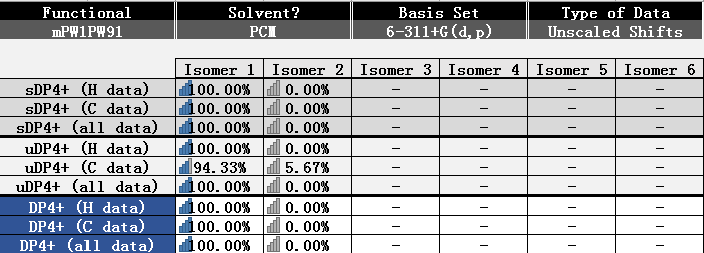


Table S4. Energy analysis and the Boltzmann Distribution for fusarisetin F (**2**).

| Conf. | G (Hartree) | ΔG (KJ/mol) | Boltzmann Distribution |
| --- | --- | --- | --- |
| 2a | | | |
| 2a1 | -1363.13582330 | 0 | 0.7834 |
| 2a2 | -1363.13367080 | 1.350699992 | 0.08 |
| 2a3 | -1363.13379100 | 1.275274144 | 0.0909 |
| 2a4 | -1363.13314100 | 1.683151029 | 0.0456 |
| 2b | | | |
| 2b1 | -1363.10936980 | 0.498237303 | 0.3012 |
| 2b2 | -1363.11016380 | 0 | 0.6988 |

Table S5. The optimized lowest energy conformers for fusarisetin F (**2**).


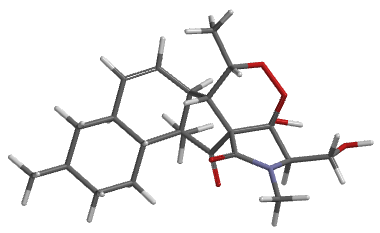

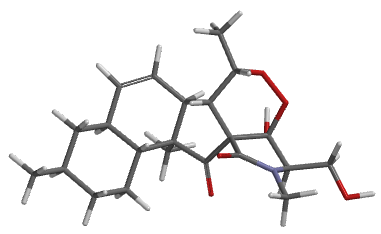

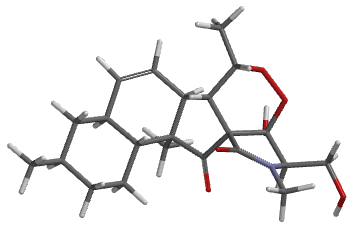


2a1 2a2 2a3


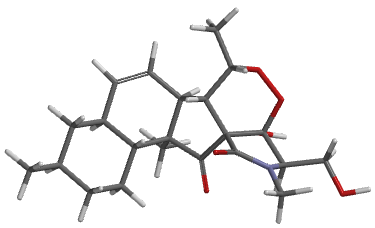

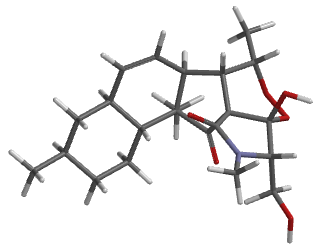

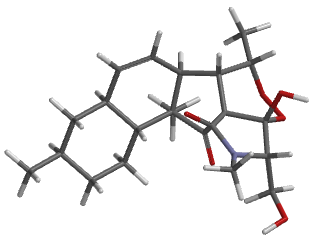


2a4 2b1 2b2

Table S6. The DP4+ evaluation of fusarisetin F (**2**).


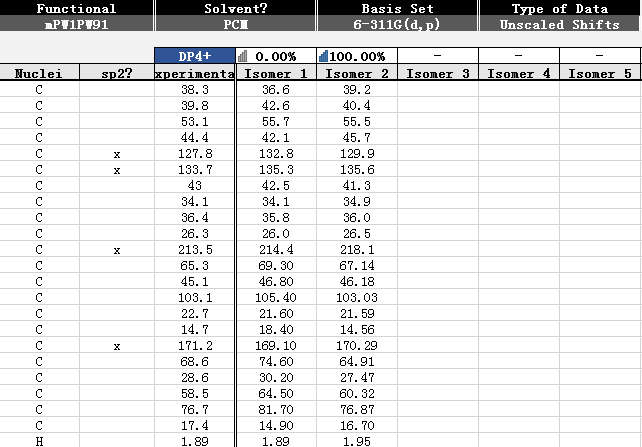


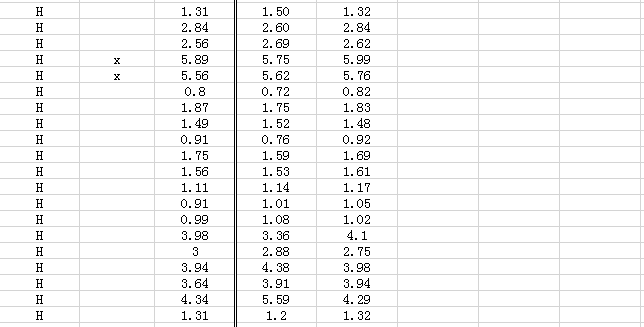


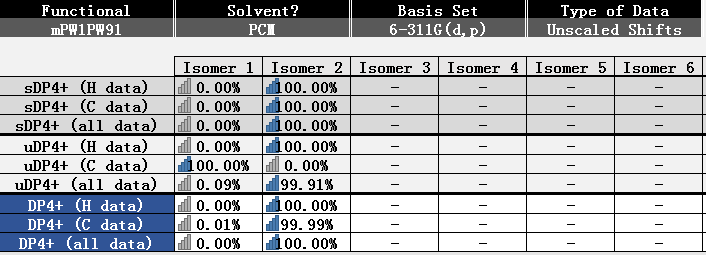


Table S7. Cartesian coordinates of fusarisetin E (**1**).

| Center  Number | Atomic  Number | Atomic  Type | Coordinates (Angstroms) | | |
| --- | --- | --- | --- | --- | --- |
|  |  |  | X | Y | Z |
| 1 | 6 | 0 | 3.413992 | 0.659488 | 0.405885 |
| 2 | 6 | 0 | 2.169284 | -0.25196 | 0.38107 |
| 3 | 6 | 0 | 0.978801 | 0.390926 | 1.184722 |
| 4 | 6 | 0 | 0.609648 | 1.765464 | 0.554586 |
| 5 | 6 | 0 | 1.822144 | 2.586193 | 0.186668 |
| 6 | 6 | 0 | 3.063838 | 2.095892 | 0.128129 |
| 7 | 6 | 0 | 4.484431 | 0.11304 | -0.56187 |
| 8 | 6 | 0 | 4.892406 | -1.32994 | -0.21539 |
| 9 | 6 | 0 | 3.648781 | -2.23412 | -0.13897 |
| 10 | 6 | 0 | 2.567168 | -1.67814 | 0.802259 |
| 11 | 6 | 0 | -0.25853 | -0.46238 | 0.922739 |
| 12 | 6 | 0 | -1.14257 | 0.179194 | -0.17412 |
| 13 | 6 | 0 | -0.35271 | 1.431448 | -0.63356 |
| 14 | 6 | 0 | -2.58848 | 0.421559 | 0.361455 |
| 15 | 6 | 0 | 5.933325 | -1.87471 | -1.19994 |
| 16 | 6 | 0 | 1.251491 | 0.48136 | 2.693509 |
| 17 | 8 | 0 | -0.51421 | -1.50967 | 1.479521 |
| 18 | 6 | 0 | -1.37752 | -0.843 | -1.29835 |
| 19 | 7 | 0 | -2.60143 | -1.41101 | -1.12125 |
| 20 | 6 | 0 | -3.39415 | -0.84523 | -0.03146 |
| 21 | 8 | 0 | -0.57432 | -1.09683 | -2.1872 |
| 22 | 6 | 0 | -3.13516 | -2.3966 | -2.04813 |
| 23 | 6 | 0 | -3.67002 | -1.80942 | 1.123888 |
| 24 | 8 | 0 | -4.59546 | -2.7827 | 0.632289 |
| 25 | 6 | 0 | -1.30293 | 2.542464 | -1.11676 |
| 26 | 6 | 0 | -0.73972 | 3.953771 | -1.23193 |
| 27 | 8 | 0 | -2.421 | 2.658408 | -0.21182 |
| 28 | 8 | 0 | -3.21377 | 1.448649 | -0.40294 |
| 29 | 8 | 0 | -2.67907 | 0.710419 | 1.727658 |
| 30 | 1 | 0 | 3.855482 | 0.613104 | 1.416909 |
| 31 | 1 | 0 | 1.840119 | -0.3142 | -0.6674 |
| 32 | 1 | 0 | 0.027273 | 2.328679 | 1.301282 |
| 33 | 1 | 0 | 0.253598 | 1.122483 | -1.49289 |
| 34 | 1 | 0 | 1.6654 | 3.642859 | -0.01222 |
| 35 | 1 | 0 | 3.890704 | 2.764254 | -0.11391 |
| 36 | 1 | 0 | 4.093572 | 0.14837 | -1.58985 |
| 37 | 1 | 0 | 5.367744 | 0.767039 | -0.5411 |
| 38 | 1 | 0 | 5.350125 | -1.30969 | 0.787169 |
| 39 | 1 | 0 | 3.224441 | -2.3454 | -1.14818 |
| 40 | 1 | 0 | 3.94216 | -3.24109 | 0.186224 |
| 41 | 1 | 0 | 1.69624 | -2.34008 | 0.800589 |
| 42 | 1 | 0 | 2.951665 | -1.67043 | 1.83169 |
| 43 | 1 | 0 | 5.526852 | -1.9155 | -2.21865 |
| 44 | 1 | 0 | 6.245121 | -2.88972 | -0.92602 |
| 45 | 1 | 0 | 6.829995 | -1.24348 | -1.22413 |
| 46 | 1 | 0 | 1.418998 | -0.50977 | 3.122238 |
| 47 | 1 | 0 | 2.123205 | 1.109965 | 2.900437 |
| 48 | 1 | 0 | 0.389528 | 0.919654 | 3.209208 |
| 49 | 1 | 0 | -4.36893 | -0.5212 | -0.42241 |
| 50 | 1 | 0 | -4.05027 | -2.01657 | -2.51915 |
| 51 | 1 | 0 | -3.37603 | -3.32554 | -1.52736 |
| 52 | 1 | 0 | -2.37588 | -2.57197 | -2.81162 |
| 53 | 1 | 0 | -4.0999 | -1.23664 | 1.955716 |
| 54 | 1 | 0 | -2.73138 | -2.26494 | 1.45418 |
| 55 | 1 | 0 | -4.69601 | -3.46009 | 1.317512 |
| 56 | 1 | 0 | -1.7167 | 2.243587 | -2.08754 |
| 57 | 1 | 0 | -1.4978 | 4.616885 | -1.65883 |
| 58 | 1 | 0 | -0.44909 | 4.353364 | -0.25578 |
| 59 | 1 | 0 | 0.137736 | 3.957484 | -1.88549 |
| 60 | 1 | 0 | -2.51646 | 1.667615 | 1.801501 |
